# Associations between the neighbourhood food environment and food and drink purchasing in England during lockdown: A repeated cross-sectional analysis

**DOI:** 10.1371/journal.pone.0305295

**Published:** 2024-07-17

**Authors:** Alexandra Irene Kalbus, Laura Cornelsen, Andrea Ballatore, Steven Cummins

**Affiliations:** 1 Department of Public Health, Environments and Society, London School of Hygiene & Tropical Medicine, London, United Kingdom; 2 Department of Digital Humanities, King’s College London, London, United Kingdom; University of Naples Federico II: Universita degli Studi di Napoli Federico II, ITALY

## Abstract

**Introduction:**

Evidence for the effect of neighbourhood food environment (NFE) exposures on diet in the UK is mixed, potentially due to exposure misclassification. This study used the first national COVID-19 lockdown in England as an opportunity to isolate the independent effects of the NFE exposure on food and drink purchasing, and assessed whether these varied by region.

**Methods:**

Transaction-level purchasing data for food and drink items for at-home (1,221 households) and out-of-home consumption (171 individuals) were available from the GB Kantar Fast Moving Consumer Goods Panel for London and the North of England. The study period included 23^rd^ March to 10^th^ May 2020 (‘lockdown’), and the same period in 2019 for comparison. NFE exposures included food outlet density and proximity, and NFE composition within a 1 km network buffer around the home. Associations were estimated for both years separately, adjusted for individual and household characteristics, population density and area deprivation. Interaction terms between region and exposures were explored.

**Results:**

There were no consistent patterns of association between NFE exposures and food and drink purchasing in either time period. In 2019, there was some evidence for a 1.4% decrease in energy purchased from ultra-processed foods for each additional 500 m in the distance to the nearest OOH outlet (IR 0.986, 95% CI 0.977 to 0.995, *p* = 0.020). In 2020, there was some evidence for a 1.8% reduction in total take-home energy for each additional chain supermarket per km^2^ in the neighbourhood (IR 0.982, 95% CI 0.969, 0.995, *p* = 0.045). Region-specific effects were observed in 2019 only.

**Discussion:**

Findings suggest that the differences in exposure to the NFE may not explain differences in the patterns or healthiness of grocery purchasing. Observed pre-pandemic region-specific effects allude to the importance of geographical context when designing research and policy. Future research may assess associations for those who relied on their NFE during lockdown.

## Introduction

The COVID-19 pandemic caused major disruption to social and public life. On 16^th^ March 2020, the UK government implemented measures aimed at minimising virus transmission by reducing social contact. These included working from home if possible and avoiding social contact through limiting non-essential travel and closing social venues such as pubs, cinemas, and theatres [1]. A week later, on 23^rd^ March 2020, nationwide rules were implemented, which are further referred to as ‘lockdown’. This consisted of the closure of all but ‘essential businesses’ such as pharmacies and supermarkets, further reducing social contact, and working and staying at home as much as possible [2]. Individuals were expected to stay at home except for limited purposes such as shopping for necessities, medical needs, exercise once a day and travel to work where absolutely necessary [2]. A staged easing of restrictions began on 11^th^ May 2020, when individuals were allowed unlimited time outdoors (including for exercise) [3]. After periods of relaxation and implementation of local as well as nationwide restrictions, most remaining legal limits on social contact were lifted on 19^th^ July 2021 [4].

Although most of the take-home food provision (supermarkets, corner stores etc.) remained open, the out-of-home (OOH) food sector, including restaurants, pubs and takeaways, was required to close (with the exception of takeaway and/or delivery services) from 23^rd^ March to 4^th^ July 2020 [5], and again in two subsequent lockdowns (5^th^ November to 1^st^ December 2020 and 6^th^ January to 7^th^ March 2021 (when a phased exit of lockdown began), respectively) [6]. A change to planning regulations enabled restaurants to switch to takeaway provision without gaining additional planning permissions [7], and subsequent takeaway consumption partly offset losses in the OOH sector during the first year of the pandemic [8].

The pandemic had a considerable impact on individual lifestyles and health behaviours, including changes in sleep, physical activity, diet, and alcohol intake [9]. Generally, grocery purchasing shifted to fewer and larger trips [10], while the use of online grocery shopping increased rapidly [11]. However, some consumers opted to use local, smaller and independent stores instead, adopting a little-but-often approach [12]. Diets were also impacted by the pandemic, with indications that fruit and vegetable intake declined [13], while consumption of sweet and savoury snacks increased [10]. Increases in alcohol consumption were also observed with one modelling study suggesting that this may lead to an additional alcohol-related 207,597 hospital admissions and 7,153 deaths by 2042 [14].

Diet and dietary health are thought to be influenced by environmental factors, including exposure to the food environment [15]. One component of the broader food environment is the neighbourhood food environment (NFE), or local food environment, which is conceptualised as the availability of, and access to physical food outlets available to residents such as supermarkets, corner stores, restaurants, and takeaway outlets around the home [16]. It is thought to influence dietary behaviour through the availability of and access to components of healthy and less healthy diets [17]. Another mechanism may be that those elements of the food environment act as environmental cues prompting behavioural responses, and/or implicitly shape norms on food choice through their composition, i.e. the relative density of different outlet types [18]. There is evidence for NFE exposure influencing dietary health outcomes including diet, body weight and obesity, as well as inequalities in these [15,19,20]. However, evidence mostly originates from the US [21]. In the UK, some studies have found associations between greater exposure to fast-food outlets and greater fast-food consumption as well as increased body weight [22,23]. Generally, however, the evidence for the relationship between the NFE and individual outcomes in the UK is mixed [24].

One potential reason for this inconsistent evidence base is misspecification of exposure, with neighbourhood studies particularly at risk of falling into the ‘local trap’ [25]: by focussing on neighbourhood food retail only, other relevant environmental exposures such as in school or work environments and the commute may be missed. Findings from previous research which considered multiple daily activity spaces indicate that this may be true [26,27]. Ill-specified exposure tends to bias estimates towards the null [28], which may further explain the inconsistent evidence base. Another factor may be the existence of geographical exposure-effect heterogeneity, whereby neighbourhood effects vary across space. That neighbourhood exposures may be more important for some people in some places than others is a common observation in neighbourhoods and health research [29–31] and alludes to the importance of contextual factors when designing research and policy interventions.

The pandemic and associated restrictions may be viewed as natural experiment, whereby reliance on the NFE as a source of grocery and takeaway purchases was increased and exposure to food outlets outside of the local residential neighbourhood (e.g. surrounding schools or workplaces) was reduced [12,32]. Food environment exposures outside the neighbourhood make it more difficult to isolate the independent effect of the NFE. Prior to the pandemic, the NFE accounted for only 30% of daily food outlet exposure experienced by UK adults [33]. Therefore, the early stages of the pandemic present a unique opportunity to better explore the independent association between the NFE and individual dietary behaviour as lockdown reduced exposure to environments outside of the local residential area. This study investigates associations between NFE exposures and food and drink purchasing outcomes during the first national lockdown in England and compares them descriptively to the pre-pandemic period. As effects of the NFE [30] and general neighbourhood [31,34] have been observed to vary by geographical context, this study’s secondary aim is to assess if geographical heterogeneity in exposure-effect associations exists by investigating if any observed associations vary by region.

## Methods

This repeated cross-sectional study builds on previous research on the relationship between the NFE and food and drink purchasing in England before the COVID-19 pandemic [35]. In this previous study, we used commercial consumer food and drink purchasing data and publicly available food outlet data to examine relationships between exposure measures capturing density, proximity and food environment composition and various take-home and OOH food and drink purchasing outcomes in 2019 [35]. The present study replicates this analysis for the period of the first national lockdown, which lasted from 23^rd^ March to 10^th^ May 2020 and is hereafter referred to ‘lockdown’. We use the same period in 2019 for comparison rather than the full year, as reported in our previous study, to rule out seasonal effects. This comparison of effects of the NFE on food and drink purchasing in 2019 and 2020 is descriptive only, as the study focusses on NFE effects during lockdown rather than how these may differ from before the pandemic.

This study used anonymised data from the consumer research company Kantar. Upon joining the panel, participants agree to the terms and conditions of the Consumer Goods Panel (see https://www.kantar.com/uki for details). Ethical approval for this study was granted by the London School of Hygiene and Tropical Medicine’s Observational Research Ethics Committee (reference number 22578).

### Data

#### Food and drink purchase data

Item-level transaction data on food and drink purchasing for in-home and OOH consumption were obtained from the Kantar Fast Moving Consumer Goods panel [36]. Kantar is a commercial research company, and households enrolled in its live consumer panel, hereafter referred to as take-home reporters, record food and drink purchases brought to the home with hand-held barcode scanners. For unbarcoded items such as loose fruit and vegetables, bespoke barcodes are provided. Kantar also collects nutritional information twice a year, which is supported by third-party supplier Brandbank and includes information about the product’s energy (kcal) content and macronutrient composition. A subsample of individuals from this take-home panel, hereafter referred to as OOH reporters, also records OOH food and drink purchases through a mobile application. For products purchased for OOH consumption, nutritional information is unknown unless purchased from supermarkets. All data used in the present study were available from The TfL Study [37], an evaluation of restrictions on unhealthy food advertisements on London’s public transport network. Available data were from the regions of Greater London and the North of England (North East, North West, and Yorkshire and the Humber).

We included food and drink purchasing data recorded during lockdown. Inclusion criteria for take-home (households) and OOH reporters (individuals) were recording purchases during lockdown and the same period of time in 2019 and residing in either London or the North of England in both years. The resulting sample sizes were n = 1,221 households in the take-home and n = 171 individuals in the OOH sample. While smaller than the samples in 2019 (2,118 take-home reporters and 447 OOH reporters), which we used in our previous study [35], the present analytical samples are similar in terms of region, household composition and socioeconomic characteristics to the full 2019 samples (see Tables A and B in S1 File). In total, our analysis included 624,153 packs of take-home food and drink items, with a ‘pack’ referring to individual products or multipacks, and 9,874 packs of products purchased for OOH consumption.

#### Food and drink purchasing outcomes

Transaction-level take-home purchase data were aggregated to the household-week level and averaged over the 7-week periods in 2019 and 2020, respectively. We then created a range of purchasing outcome measures described as follows. Frequency of purchasing was defined as number of days per week with purchase occasions. Total energy purchased was defined as the average weekly energy (kcal) purchased per household member. Energy that households purchased from (i) fruits and vegetables, (ii) foods and drinks high in fat, salt and sugar (HFSS), and (iii) ultra-processed foods (UPF), were expressed as a proportion of total energy purchased. Fruits and vegetables were defined based on a previously developed classification [38]. Products were classified as HFSS according to the Nutrient Profiling Model (NPM) [39] as previously described [40]. In brief, an item’s energy, sugar, salt, and saturated fat content was weighed against its protein, fibre, and fruit and vegetable content to calculate a score, with higher values indicating that a product is less healthy. Food products that scored ≥ 4 points and drink products that scored ≥ 1 point were classified as HFSS [41]. UPF were defined according to the NOVA classification [42] which was applied to Kantar’s proprietary product classifications. Both HFSS and UPF classifications were used in this study, even though there is overlap. Classification of HFSS products is based on macronutrient composition and has been used in a number of policies in the UK such as advertisement or product placement restrictions [43]. However, HFSS food and drink consumption has not consistently been associated with health outcomes [44]. The NOVA classification, on the other hand, focuses on the level of processing. UPF consumption has been associated with adverse dietary health [45], but this classification is yet to be used in policy making. Alcohol purchases were expressed as volume (ml) of alcoholic beverages per week and adult in the household. Nutritional information was not available for OOH purchases. Therefore, we calculated the frequency of OOH purchasing as the average number of days with OOH purchasing occasions per 28-day period, referred to as ‘month’.

#### Neighbourhood food environment data

The smallest geography available was the postcode district of residence. The geography of postcodes is primarily used by the main UK postal service, Royal Mail, to determine delivery areas [46]. The first half of a postcode is a postcode district, for example, ‘NW3’. In our study sample, households were distributed over 553 postcode districts with a median size of 14.72 km^2^ (interquartile range 6.71 to 36.24) and a median population of 33,387 (IQR 23,725 to 44,423) in 2020. We assumed that the most likely household location corresponds to the point closest to most of the resident population within a postcode district. Therefore, we assigned each household to the population-weighted centroid of its postcode district of residence. We defined the ‘neighbourhood’ as 1 km street network buffer around this centroid using ArcGIS Online. This neighbourhood equates to a 15-minute walk and is commonly used in NFE research [47,48]. Postcode district boundaries were obtained from the University of Edinburgh’s DataShare Service [49].

NFE exposure data were obtained from Ordnance Survey Points of Interest (POI) for March 2019 and March 2020 under an educational licence [50] and categorised into ‘supermarkets’ and ‘OOH outlets’. Supermarkets included independent and chain supermarkets and convenience stores and were classified using a name-based approach according to Table 1. OOH outlets were categorised into ‘restaurants’ and ‘takeaway outlets’ as previously described [35]. In brief, historical POI data were assigned policy-relevant definitions of food outlets by cross-referencing them against Food Hygiene Rating Scheme (FHRS) data published by the Food Standards Agency [51,52].

**Table 1:** Classification of supermarkets.

Classification	Outlet description
Chain supermarkets	Supermarket chains (e.g. Tesco, Morrisons, Waitrose) and convenience symbol groups (e.g. Nisa, Co-op, Costcutter)
Independent supermarkets	Food retailers comprising less than 5 outlets in POI data
All supermarkets	Chain supermarkets and independent supermarkets
excluded	Outlets selling primarily non-food items (e.g. newsstands) and outlets located in service stations

#### Neighbourhood food environment exposures

Three types of NFE exposures were created: distance, density and composition measures. These represent absolute measures of proximity and availability, and a relative measure of food environment composition, which are commonly used in NFE research [53]. The distance from the inferred household address to the nearest food outlet along the road network was determined using ArcMap version 10.5 and Ordnance Survey Open Roads [54]. Food outlet density was calculated as count of respective outlets in the neighbourhood divided by its area (km^2^). The composition measure compared densities of supermarkets and OOH outlets in a neighbourhood. Accordingly, a neighbourhood either had a greater number of supermarkets, a greater number of OOH outlets, or no outlets.

#### Covariates

Household sociodemographic characteristics included in this analysis were age (in years), sex, and social grade according to the National Readership Survey (NRS) of the main reporter, and the number of adults and children (< 16 years) in the household. The NRS defines social grade based on occupation and includes the categories AB “Higher and intermediate managerial, administrative and professional”; C1C2 “Supervisory, clerical and junior managerial, administrative and professional; and Skilled manual workers”, and DE “Semi-skilled and unskilled manual workers; and State pensioners, casual and lowest grade workers, unemployed with state benefits only” [55]. Region of residence (London or North of England) was also included.

Population estimates for 2019 and 2020 were retrieved from the Office for National Statistics [56] and interpolated from the Lower Layer Super Output Area (LSOA) to the postcode district level using extensive area interpolation [57]. Population density in the postcode district was expressed as population per km^2^. We defined area deprivation as the income deprivation domain of the Index of Multiple Deprivation England [58]. We interpolated income scores from the LSOA to postcode district level using intensive area interpolation, and then ranked postcode districts according to their income deprivation score [59].

#### Analytical sample

To address potential underreporting, periods of two or more consecutive weeks of non-reporting were removed from the take-home purchase data, in line with previous reported work [8]. With respect to OOH purchasing, weeks were removed if they coincided with the household underreporting take-home purchases. OOH purchases recorded not by the main OOH reporter but another household member were excluded, as no individual characteristics of those reporters were known.

### Statistical analysis

If not otherwise specified, all data management and analysis tasks were performed with R version 4.1.3. Alpha was determined at 0.05. The two years were analysed separately, and results were compared descriptively.

Sample description was followed by bivariate explorations of associations between purchase outcomes and NFE exposures in both years. Global Moran’s I was calculated using GeoDa software to test for spatial autocorrelation (see Table C in S2 File). None was detected, and we carried the multivariable analysis out without accounting for spatial dependency. Because the outcomes were over-dispersed count data, negative binomial models were used, and model choice was guided by the Bayesian Information Criterion (BIC) and Root Mean Square Error (RMSE). Accordingly, non-hierarchical negative binomial models fitted the data best for all outcomes. For food outlet density and distance measures, we explored if these exposures were best modelled as numeric or categorical variables and compared the fit of models with the respective variables as numeric indicators and split into tertiles and quartiles. BIC and RSME were consistently better for numeric expressions of density and distance exposures, and those were modelled. Because food and drink purchasing outcomes were expressed as rates, e.g. total energy purchased per week and household member, we modelled respective offsets, i.e. log terms with a coefficient of 1.

All models adjusted for age, sex and social grade of the main shopper, number of adults and children in the household, region, population density, and area deprivation. To reflect the diversity between the study regions, interactions between region and social grade of the main shopper, population density and area deprivation were modelled. We modelled each NFE exposure measure separately. As shown in Table 2, we modelled aggregated OOH outlet exposure for take-home purchasing outcomes, and vice versa, we used aggregated supermarket exposure for OOH purchasing. We scaled distance measures to a 500 m difference to ease interpretation of coefficients.

**Table 2:** Neighbourhood food environment exposures examined in models for take-home and out-of-home purchasing.

Take-home purchasing models	Out-of-home purchasing models
Density of chain supermarkets (count/km^2^)	Density of all supermarkets (count/km^2^)
Distance to nearest chain supermarket (m)	Distance to nearest supermarket (any) (m)
Density of independent supermarkets (count/km^2^)	Density of restaurants (count/km^2^)
Distance to nearest independent supermarket (m)	Distance to nearest restaurant (m)
Density of OOH outlets (count/km^2^)	Density of takeaway outlets (count/km^2^)
Distance to nearest OOH outlet (m)	Distance to nearest takeaway outlet (m)
Composition of the food environment • More supermarkets • More OOH outlets • No outlets	Composition of the food environment • More supermarkets • More OOH outlets • No outlets

We addressed multiple testing by adjusting *p* values according to the Benjamini-Hochberg approach [60]. This method controls the false-discovery rate, i.e. the expected proportion of rejecting the null hypothesis when in fact it was true (type I error) and involves adjusting *p* values according to their rank within the set of tests. Hence, all hypotheses following the first to be rejected after *p*-value adjustment will also be rejected. This method has higher power compared to methods controlling the family-wise error rate such as the Bonferroni correction [60]. To determine the family of tests, we treated each outcome and each year as independent from each other.

#### Secondary analysis

We examined region-specific associations between NFE exposures and purchasing by modelling an additional interaction term between region and the respective NFE exposure.

#### Sensitivity analysis

We examined robustness of observed results regarding the density measures’ buffer size, definition of supermarkets, inclusion of OOH purchases not recorded by the main reporter, and exclusion of take-home purchases made online. We explored buffers of 0.5 km, 2 km and 5 km to assess if the chosen (1 km) neighbourhood delineation affects results. We assessed if the chosen aggregation of grocery retailers affected results by exploring exposure to big chain supermarkets, small chain supermarkets and convenience symbol groups, and independent supermarkets separately. Furthermore, we analysed all OOH purchases, including those reported by household members for whom sociodemographic characteristics were unknown. Finally, we excluded all take-home purchases made online, because online grocery delivery may mask the relationship between the NFE and food and drink purchasing. This led to the exclusion of 20 households in 2019 and 25 in 2020 who exclusively reported online food and drinks purchases. A total of 552,782 packs of food and drink items purchased in-store were included in this sensitivity analysis, corresponding to 88.57% of all packs.

## Results

Table 3 shows the socio-demographic characteristics of the take-home and OOH reporters. Tables 4 and 5 display descriptive statistics for area characteristics and purchasing outcomes for the take-home and OOH reporters stratified by year, respectively. Of the 1,221 households reporting take-home purchases, most resided in the North of England (56.8%), consisted of two adults (38.1%) and had no children (74.4%). Main shoppers were predominantly female (71.7%), had a median age of 54 years and were of social grade C1C2 (60.2%). OOH reporters (n = 171) were mostly similar to take-home reporters, but somewhat younger with a median age of 49 years, and relatively more OOH reporters resided in the North of England (60.2%) compared to take-home reporters.

**Table 3:** Sample characteristics. Median (IQR) and n (%).

	Take-home reporters (n = 1,221)	OOH reporters (n = 171)
Region		
London	527 (43.16)	68 (39.77)
North of England	694 (56.84)	103 (60.23)
Age of main shopper	54 (44, 64)	49 (42, 58)
Gender of main shopper		
Female	875 (71.66)	120 (70.18)
Male	346 (28.34)	51 (29.82)
NRS social grade of main shopper		
AB	216 (17.69)	29 (16.96)
C1C2	735 (60.20)	109 (63.74)
DE	270 (22.11)	33 (19.30)
Number of people in the household		
1	262 (21.46)	30 (17.54)
2	465 (38.08)	73 (42.69)
3	219 (17.94)	32 (18.71)
4	206 (16.87)	29 (16.96)
5+	69 (5.65)	7 (4.09)
Children in the household		
Yes	313 (25.63)	48 (28.07)
No	908 (74.37)	123 (71.93)

IQR = interquartile range; NRS = National Readership Survey [55].

**Table 4:** Description of area characteristics and outcome variables over time, take-home reporters (n = 1,221). Median (IQR) and n (%).

	2019	2020
Population density (people/km^2^)	2,908 (1,195, 5,288)	2,922 (1,228, 5,376)
Density of chain supermarkets (outlets/km^2^)	2.56 (1.21, 3.87)	2.68 (1.25, 4.17)
Density of independent supermarkets (outlets/km^2^)	1.73 (0.59, 5.41)	1.73 (0, 5.57)
Distance to nearest chain supermarket (m)	538.62 (323.27, 895.49)	533.07 (323.27, 893.13)
Distance to nearest independent supermarket (m)	674.41 (341.51, 1,095.95)	687.30 (346.91, 1,123.55)
Density of OOH outlets (outlets/km^2^)	7.69 (2.58, 17.89)	8.16 (2.61, 18.88)
Distance to nearest OOH outlet (m)	494.81 (264.41, 787.39)	473.87 (259.32, 796.70)
Food environment composition		
More supermarkets	283 (23.18)	277 (22.69)
More OOH outlets	807 (66.09)	815 (66.75)
No outlets	131 (10.73)	129 (10.57)
Frequency (days)	1.86 (1.14, 2.57)	1.43 (1.00, 2.14)
Total kcal (kcal) ^a^	11,139.40 (7,823.86, 14,767.55)	13,171.43 (9,791.92, 17,115.78)
kcal from fruit & vegetables (%)	3.93 (2.49, 6.25)	3.53 (2.33, 5.21)
kcal from HFSS products (%)	52.31 (45.14, 59.06)	53.55 (46.10, 59.25)
kcal from UPF (%)	59.24 (49.61, 68.68)	57.36 (47.61, 67.07)
Volume of alcohol (ml) ^b^	89.29 (0, 535.71)	160.71 (0, 836.67)

Values are percentages for categorical variables and median (interquartile range) for continuous variables. IQR = interquartile range; OOH outlets = outlets for out-of-home consumption, include restaurants and hot food takeaways; HFSS = high in fat, salt and sugar (according to the Nutrient Profiling Model [41]); UPF = ultra-processed foods (according to the NOVA classification [42]).

^a^ per household member and week.

^b^ per adult and week.

**Table 5:** Description of area characteristics and outcome variables over time, OOH reporters (n = 171). Median (IQR) and n (%).

	2019	2020
Population density (people/km2)	3,172.47 (1,389.61, 5,409.90)	3,210.87 (1,388.82, 5,514.64)
Density of supermarkets (outlets/km^2^)	5.13 (2.25, 11.46)	5.13 (2.07, 11.70)
Distance to nearest supermarkets (m)	397.56 (196.38, 689.42)	382.24 (188.26, 695.14)
Density of restaurants (outlets/km^2^)	3.20 (0.61, 10.90)	3.67 (0.75, 11.33)
Distance to nearest restaurant (m)	544.55 (330.24, 945.63)	536.60 (307.35, 913.11)
Density of takeaway outlets (outlets/km^2^)	4.41 (1.48, 8.67)	4.66 (1.48, 9.35)
Distance to nearest takeaway outlet (m)	495.62 (266.44, 844.22)	473.18 (262.16, 869.06)
Food environment composition		
More supermarkets	35 (20.47)	32 (18.71)
More OOH outlets	117 (68.42)	120 (70.18)
No outlets	19 (11.11)	19 (11.11)
Purchasing frequency (days/month)	4.57 (2.86, 10.00)	1.71 (1.14, 4.00)

Values are percentages for categorical variables and median (interquartile range) for continuous variables. IQR = interquartile range; OOH outlets = outlets for out-of-home consumption, include restaurants and hot food takeaways.

In 2020, exposure to OOH outlets was greater than exposure to supermarkets, with two thirds of neighbourhoods having more OOH outlets than supermarkets (66.8% and 70.2% among take-home and OOH reporters, respectively). No food outlets were present in 10.6% of neighbourhoods among take-home reporters, and 11.1% among OOH reporters. Overall exposure to the NFE was greater in London compared to the North of England. NFE exposure was similar in both years, with slightly higher exposure to OOH outlets in 2020 compared to 2019 (e.g. take-home reporters: 66.1% and 66.8% have more OOH outlets in neighbourhood in 2019 and 2020, respectively; OOH reporters: 68.4% and 70.2% have more OOH outlets in their neighbourhood in 2019 and 2020, respectively).

During lockdown, households purchased food and drinks for take-home consumption on a median of 1.4 days per week, which was lower than the same period in 2019 (1.9 days/week). Median purchased energy from foods and drinks brought to the home increased to 13,171 kcal per household member per week, compared to 11,139 kcal in 2019. Of the purchased energy, 3.9% was from fruits and vegetables (3.5% in 2019), 53.6% from HFSS foods and drinks (52.3% in 2019), and 57.4% from UPF (59.2% in 2019). The median weekly volume of purchased alcoholic beverages for at-home consumption was 160.7 ml per adult, compared to 89.3 ml in 2019. Individuals reported OOH purchases on a median 4.2 days per month, which was lower than in 2019 (4.6 days per month).

Bivariate analysis showed that more deprived and more densely populated areas were associated with greater exposure to food outlets. Tables D–G in S3 File, contains the full bivariate analysis.

### Associations between neighbourhood food environment exposures and purchases

Some evidence of a relationship between neighbourhood food environment exposures and food and drink purchasing both in 2019 and 2020 was observed in the bivariate analysis (see Tables D and E in S3 File). However, the multivariable analysis (Tables 6 and 7) after adjustment for multiple testing did not provide evidence for a consistent relationship in either year. Magnitude and direction of relationships were broadly consistent across the two years. During lockdown, there was some evidence for a relationship between the distance to chain supermarkets and purchasing frequency, as well as between the density and chain supermarkets and total take-home energy purchased. For each additional 500 m in distance to the nearest chain supermarket, food and drink purchasing frequency decreased by 2.3% (IR 0.978, 95% CI 0.963 to 0.994, *p* = 0.050). Weekly household energy purchased decreased by 1.8% for each additional chain supermarket per km^2^ in the household’s neighbourhood (IR 0.982, 95% CI 0.969 to 0.995, *p* = 0.045).

**Table 6:** Parameter estimates and 95% CI of take-home purchase outcomes associated with neighbourhood food environment exposures.

		Frequency (days)			Total energy (kcal)			Energy from fruit & vegetables (kcal)			Energy from HFSS products (kcal)			Energy from UPF (kcal)			Alcohol volume (ml)		
Exposure	Year	IR	95% CI	*p* value	IR	95% CI	*p* value	IR	95% CI	*p* value	IR	95% CI	*p* value	IR	95% CI	*p* value	IR	95% CI	*p* value
Density of chain supermarkets	2019	1.002	0.986, 1.018	0.908	0.996	0.983, 1.010	0.635	0.997	0.976, 1.020	0.981	1.005	0.998, 1.012	0.310	1.005	0.997, 1.014	0.267	0.962	0.860, 1.076	0.922
	2020	0.995	0.980, 1.011	0.614	0.982	0.969, 0.995	0.045	0.991	0.971, 1.010	0.565	1.003	0.997, 1.010	0.737	1.001	0.992, 1.009	0.893	0.956	0.869, 1.052	0.894
Distance to chain supermarkets	2019	0.987	0.972, 1.003	0.287	1.008	0.995, 1.021	0.632	1.015	0.993, 1.036	0.473	0.992	0.985, 0.999	0.177	0.990	0.982, 0.998	0.048	1.020	0.921, 1.130	0.922
	2020	0.978	0.963, 0.994	0.050	1.000	0.987, 1.013	0.993	1.018	0.999, 1.038	0.180	0.997	0.991, 1.004	0.737	0.992	0.983, 1.000	0.137	0.965	0.879, 1.058	0.894
Density of independent supermarkets	2019	1.000	0.991, 1.008	0.908	0.998	0.991, 1.005	0.635	1.000	0.988, 1.011	0.981	0.999	0.995, 1.002	0.554	1.000	0.996, 1.005	0.883	0.968	0.916, 1.022	0.922
	2020	0.994	0.986, 1.002	0.328	0.995	0.988, 1.001	0.320	1.000	0.990, 1.011	0.974	0.999	0.995, 1.002	0.737	0.997	0.993, 1.002	0.265	0.994	0.948, 1.042	0.894
Distance to independent supermarkets	2019	0.991	0.976, 1.006	0.352	1.010	0.997, 1.023	0.500	1.009	0.988, 1.030	0.802	0.997	0.990, 1.003	0.419	0.992	0.985, 0.999	0.070	0.997	0.892, 1.113	0.953
	2020	0.993	0.978, 1.009	0.549	1.005	0.992, 1.018	0.815	1.012	0.992, 1.033	0.450	0.996	0.990, 1.003	0.737	0.994	0.985, 1.002	0.265	0.980	0.891, 1.078	0.894
Density of OOH outlets	2019	1.002	0.999, 1.004	0.352	0.999	0.997, 1.001	0.635	1.000	0.996, 1.004	0.981	1.000	0.999, 1.001	0.841	1.000	0.998, 1.001	0.883	0.996	0.979, 1.014	0.922
	2020	1.001	0.999, 1.004	0.439	0.998	0.995, 1.000	0.103	0.999	0.996, 1.002	0.728	1.000	0.999, 1.001	0.797	0.998	0.997, 0.999	0.056	0.999	0.983, 1.015	0.894
Distance to OOH outlets	2019	0.982	0.964, 1.001	0.287	1.008	0.992, 1.024	0.632	1.022	0.996, 1.048	0.373	0.993	0.985, 1.001	0.267	0.986	0.977, 0.995	0.020	1.015	0.901, 1.143	0.922
	2020	0.983	0.965, 1.002	0.322	0.997	0.982, 1.013	0.815	1.029	1.005, 1.053	0.130	0.994	0.986, 1.001	0.737	0.989	0.979, 0.999	0.126	0.971	0.866, 1.089	0.894
Food environment composition																			
More OOH outlets	2019	0.994	0.926, 1.068	0.908	0.997	0.938, 1.059	0.913	1.013	0.918, 1.118	0.981	0.979	0.949, 1.010	0.310	0.973	0.938, 1.009	0.218	1.077	0.672, 1.727	0.922
	2020	0.998	0.929, 1.072	0.957	0.987	0.292, 1.049	0.815	0.996	0.909, 1.092	0.974	1.007	0.977, 1.038	0.797	0.973	0.936, 1.012	0.265	1.214	0.782, 1.884	0.894
No outlets	2019	0.906	0.811, 1.012	0.287	1.075	0.980, 1.179	0.500	1.150	0.990, 1.337	0.373	0.960	0.915, 1.008	0.267	0.940	0.889, 0.993	0.070	1.349	0.653, 2.788	0.922
	2020	0.935	0.838, 1.042	0.439	1.029	0.938, 1.129	0.815	1.149	0.999, 1.321	0.180	1.003	0.958, 1.050	0.900	0.967	0.911, 1.026	0.303	1.298	0.659, 2.557	0.894

95% CI = 95% confidence interval; HFSS = high in fat, salt and sugar; IR = Incidence Rate; OOH = out of home; UPF = ultra-processed foods. Effect estimates of density measures refer to a change in incidence rate in response to an increase of 1 outlet/km^2^. Effect estimates of distance measures refer to a change in incidence rate in response to an increase of 500 m. The reference category for the composition of food environments is neighbourhoods with more supermarkets.

All models are adjusted for age, sex and social grade of the main shopper, number of children and adults in the household, region, area deprivation and population density, and interactions between region and social grade, area deprivation, and population density. *p* values were adjusted for multiple testing using the Benjamini-Hochberg method.

**Table 7:** Parameter estimates and 95% CI of OOH purchasing frequency associated with neighbourhood food environment exposures.

	2019			2020		
Exposure	IR	95% CI	*p* value	IR	95% CI	*p* value
Density of all supermarkets	0.969	0.940, 0.999	0.141	0.975	0.940, 1.011	0.541
Distance to any supermarket	0.911	0.813, 1.020	0.214	0.914	0.796, 1.050	0.541
Density of restaurants	0.982	0.964, 1.000	0.141	0.992	0.970, 1.014	0.808
Distance to restaurants	0.966	0.898, 1.038	0.396	0.990	0.907, 1.080	0.932
Density of takeaway outlets	0.987	0.957, 1.018	0.406	0.992	0.956, 1.029	0.870
Distance to takeaway outlets	0.957	0.897, 1.021	0.287	0.997	0.921, 1.079	0.938
Composition of food environments						
More OOH	0.856	0.620, 1.182	0.396	1.331	0.882, 2.010	0.541
No outlets	0.552	0.335, 0.911	0.141	0.810	0.436, 1.505	0.808

95% CI = 95% confidence interval; OOH = out of home; IR = Incidence Rate. Effect estimates of density measures refer to a change in incidence rate in response to an increase of 1 outlet/km^2^. Effect estimates of distance measures refer to a change in incidence rate in response to an increase of 500 m. The reference category for the composition of food environments is neighbourhoods with more supermarkets.

All models are adjusted for age, sex, NRS social grade, number of children and adults in the household, region, area deprivation and population density, and interactions between region and NRS social grade, area deprivation, and population density. *p* values were adjusted for multiple testing using the Benjamini-Hochberg method.

In 2019, there was some evidence for a relationship between purchasing of energy from take-home UPF and the distance to chain supermarkets and OOH outlets. Accordingly, an increase of 500 m in the distance to the nearest chain supermarket was associated with a reduction of 1.0% in energy purchased from UPF (incidence rate 0.990, 95% confidence interval 0.982 to 0.998, *p* = 0.048), while an additional 500 m in the distance to the nearest OOH outlet was associated with a decrease of 1.4% in energy purchased from UPF (IR 0.986, 95% CI 0.977 to 0.995, *p* = 0.020).

### Secondary analysis

Results of the region-specific analyses can be found in Tables H–K in S4 File. We did not observe interactions between NFE exposure and region on food and drink purchasing outcomes during lockdown. In 2019, region moderated the associations between the distance to chain supermarkets and purchasing frequency, and between the food environment composition and total energy purchased. Despite the interaction, there was no effect of distance to chain supermarkets on purchasing frequency in either region. In both regions, the absence of food outlets in the neighbourhood was associated with increased total energy purchased, but this association was stronger in London. Households living in London neighbourhoods without food outlets had 55% greater energy purchases compared to those living in neighbourhoods with more supermarkets than OOH outlets (IR 1.547, 95% CI 1.261 to 1.897, *p*<0.001). Households in the North of England living in neighbourhoods without food outlets purchased 22% higher energy compared to those living in neighbourhoods with more supermarkets than OOH outlets (IR 1.224, 95% CI 1.092 to 1.373, *p* = 0.004). There was no effect modification by region observed for the other purchasing outcomes.

It is further worth noting that in the region-specific analysis, the effects observed in the main analysis were not present. The exception is the association between the density of chain supermarkets and total energy purchased in 2020: In the North of England, a higher density of chain supermarkets was associated with 1.8% lower total energy purchased in 2020 (IR 0.982, 95% CI 0.969 to 0.994, p = 0.042). In London however, this association did not remain statistically significant after *p*-value adjustment.

### Sensitivity analysis

Sensitivity analyses (see Tables L–P in S5 File) revealed that results were mostly robust to the choice of buffer size, with similar size and magnitude of effect across buffer sizes. Despite some discrepancies between the chosen 1 km buffer and the ones explored in the sensitivity analysis (0.5, 2, and 5 km), results generally remained non-significant and were in no apparent relationship with the chosen buffer size. Observed associations were robust to the aggregation of supermarket definitions, with similar effect magnitudes and directions across the varying classifications. The inclusion of all OOH purchases instead of only those from the main reporter led to similar results, suggesting that household OOH purchasing was similar to the main reporter’s purchasing frequency in relation to NFE characteristics. Finally, the exclusion of take-home purchases made online led to similar findings in that there were no consistent patterns of association. Although the direction and magnitude of effects were similar to those observed in the main analysis, the only association that remained statistically significant after adjustment for multiple testing was between the distance to OOH outlets and energy purchased from UPF (IR 0.986, 95% CI 0.977 to 0.996, *p* = 0.049) in 2019, which may be due to lower power as a consequence of the smaller sample size compared to the main analysis.

## Discussion

### Summary of findings

This study, using large-scale objectively collected consumer purchase data, aimed to explore associations between NFE exposure and food and drink purchasing in England during the first national lockdown, and whether these varied by region and differ from the pre-pandemic period. We did not observe consistent patterns of association in 2019 and 2020. During lockdown, there was evidence of associations between frequency of take-home food and drink purchasing and distance to chain supermarkets as well as between total take-home energy purchased and the distance to chain supermarkets. For the pre-pandemic period, we observed associations between purchasing of take-home energy from UPF and the distance to chain supermarkets and OOH outlets. Limited evidence for region-specific effects was found for 2019.

### Interpretation of findings

This analysis, though not its primary focus, found considerable changes in food and drink purchasing during lockdown compared to the same period in 2019, including increases in overall household purchases of energy as well as volume of alcohol purchased for at-home consumption. However, these changes were not found to be related to the NFE, as no consistent associations between NFE exposure and purchasing outcomes were observed during lockdown and in 2019. This is in line with prior research from the UK, where evidence on the relationship between the local food environment and individual outcomes is mixed [24]. For instance, an analysis using data from the Yorkshire Health Study reported inconsistent associations between neighbourhood fast-food outlet exposure and obesity [61]. By way of contrast, a study using data from three UK diabetes screening studies found positive associations between neighbourhood fast-food outlet exposure and diabetes and obesity risk [62].

There are several possible reasons for the absence of consistent patterns of association between NFE exposure and food and drink purchasing outcomes observed in this study. First, residual confounding cannot be ruled out in the present study [48]. Second, the study may have been underpowered to detect small effects. Evidence on relationships between the NFE and dietary outcomes typically involves small effect sizes and originates from well-powered studies [63]. Third, correction for multiple testing may have resulted in Type II error, where a null hypothesis was not rejected when in fact it should have been, and as a result, some associations may have been missed in this study. However, due to the multiple exposure-outcome associations tested in this study (8 exposures x 7 outcomes each in 2019 and 2020), results were at risk of Type I error of rejecting the null hypothesis when in fact it was true, warranting adjustment [64]. The Benjamini-Hochberg method employed in this study has higher statistical power than other adjustments such as the Bonferroni correction [65]. Fourth, due to the ubiquitous availability of grocery and OOH outlets in the UK, especially in urban centres [66,67], remaining heterogeneity in NFE exposure may not suffice to drive differences in food purchasing behaviour. Finally, pandemic-related restrictions may have affected purchasing behaviour in ways that mitigated the impact of NFE exposure. For instance, as on-premises consumption was not permitted during lockdown, restaurants in the neighbourhood may not have been open at all and therefore did not constitute a true exposure, particularly during the first weeks before establishing a takeaway business. As it was unknown which OOH food outlets were operating at which time, we included all outlets as exposures assuming that even temporarily closed outlets may still constitute implicit norms [18]. Grocery shopping was perceived as challenging during this time too due to restrictions and fear of contracting COVID-19 [12]. Hence, a common change in purchasing was to opt for less frequent and larger grocery shopping trips [10]. Especially for households with access to a car, these were often realised through visiting bigger supermarkets outside urban centres and further away from their home [12].

Notwithstanding these considerations, it is also possible that there is no relationship between exposure to the NFE and individual food and drink purchasing outcomes in the UK. The lockdown can be viewed as a natural experiment: most individuals were confined to their homes and consequently, their NFE for seven weeks. Exposure to food environments outside the home, including work and school settings as well as along transport routes was both speculated and investigated as potentially biasing factors in prior research [24,68,69]. During lockdown, exposure to non-residential food environments was ruled out for most individuals. If there was a true and meaningful relationship between the NFE and individual behaviour, there would have been a greater chance that this would have been revealed in this analysis. There was some indication that effects were stronger during lockdown (see Tables 6 and 7), but differences were very small and likely due to chance.

The region-specific effects observed in this study allude to the importance of geographical context. The studied regions are different, with London different from the rest of England with respect to its population, economy, culture, and built environment [70–72]. As such, it would be reasonable to assume that exposure to elements of the food environment, alongside other environmental factors, may have different effects on individuals in different geographical contexts. Further, it is worth noting that effect modification by region was only present in 2019. During lockdown, associations between exposure to the NFE and food and drink purchasing were similar in both studied regions. This finding may suggest that the lockdown removed regional diversity to an extent, including influences on purchasing behaviour that are specific to the geographical context. As a result, the relationship between the NFE and purchasing outcomes was uniform across space. If true, the lockdown helped crystallise this relationship. On the other hand, it may be that other individual and contextual factors not captured in this study moderated the association between NFE exposure and individual food and drink purchasing.

Further, the mixed evidence on the relationship between NFE exposure and dietary health outcomes in the UK suggests that a universal pattern of association is unlikely, but there may well be geographical heterogeneity in exposure-outcome associations. Thereby, associations are affected by wider contextual factors and important effects in places which are more sensitive to environmental factors than others may be masked by average, population-wide estimates [29]. Using data from the UK Biobank, Mason and colleagues for instance showed that the association between fast-food outlet exposure and BMI varied across space in urban England [73]. Geographical exposure-effect heterogeneity could explain why national studies produce less consistent evidence on the relationship between the NFE and dietary health outcomes than studies investigating one geographical setting. In the present study, geographical heterogeneity resulted in some relationships only observed in one of the studied regions, while some associations were masked by global estimates in the main analysis. However, region-specific estimates also did not suggest stronger associations during lockdown.

Qualitative research by Thompson and colleagues on changing food purchasing behaviours in East England during the COVID-19 pandemic revealed two trends [12]: Some individuals stayed local, either because they actively chose and supported their residential food environment, or because they were restricted to it [12]. Others however did not rely on their local food environment, as they chose to drive out to bigger supermarkets further away from their home to frequent potentially better-stocked stores with fewer customers, and/or utilised online grocery shopping [12]. In our study, we do not know the location of transactions, and therefore could not determine if households and individuals stayed local. While at the population level, the NFE was not associated with food and drink purchasing in this study, the global effect estimates may have masked important relationships within those who relied exclusively on their local food environment during lockdown.

Online grocery shopping as well as delivery of meals prepared away from home increased during the pandemic [11,74]. To assess potential bias through purchases made online, we restricted the analysis of take-home purchases to those made in physical outlets in the sensitivity analyses. Using these restricted data, for neither year did we observe stronger associations as would be expected if there was a true relationship between the NFE and food and drink purchasing outcomes which was obscured by online purchases.

### Implications for research and policy

The pandemic was associated with changes in food and drink purchasing which may translate into changes in diet quality and subsequent health outcomes. While some changes may have been short-lived, there is evidence that others persisted: For instance, total energy purchased was higher not only during lockdown as observed in the current study, but throughout the remainder of 2020, as found by O’Connell and colleagues [8]. Modelling by the same group suggests that even if purchased energy decreased to pre-pandemic levels in 2021, overweight would have increased by 5% [8]. Purchases of alcoholic beverages were also higher during lockdown compared to 2019, which was partly explained through offsetting consumption that would have taken place in the OOH sector [75]. However, alcohol consumption during the pandemic increased in those who were already at-risk drinkers [76,77]. Consequently, alcohol-related premature mortality in 2020 was up 20% compared to 2019 and mainly driven by alcoholic liver disease [77]. Further, modelling suggests that there may be between 2,431 and 9,914 additional premature alcohol-related deaths in England by 2035 [78]. These worrying trends need to be closely monitored.

The outlined changes in food and drink purchasing during the pandemic do not appear to be related to the NFE. It may be that the present study missed effects of the food environment on those who relied on their NFE during the pandemic, which were masked at the population level as individuals may have opted to leave their NFE and/or use online grocery shopping and meal delivery services [12]. Therefore, future research may address the relationships between the NFE and food and drink purchasing as well as subsequent dietary health outcomes explicitly in those who stayed local in their food and drink procurement during lockdown.

Other elements of the food environment may be more relevant to individual dietary health outcomes than the neighbourhood. Such include the school and work food environment, whose cumulative exposure with the NFE has been shown to affect dietary outcomes more strongly than each independent exposure alone [17,22]. Taxation and advertising restrictions have also been shown to influence dietary choices, with two recent successful UK implementations being the Soft Drinks Industry Levy [79] and the restriction of advertising HFSS products in London’s public transport network [40]. The potential of such successful interventions should be harnessed by expanding respective programmes rather than focusing efforts on the NFE. The neighbourhood may still be a useful intervention setting in areas where there is evidence of associations between NFE exposure and dietary health. The geographical heterogeneity observed both this study and previously [35,73] suggests that effects are unlikely to be universal and both research and policy interventions should be context-specific.

### Strengths and limitations

This study has several strengths as follows. Firstly, this study took advantage of granular and objectively recorded food and drink purchasing data. Recorded purchase data have a lower risk of bias than outcome measures which rely on participants’ memory such as diet recalls [80]. These data also enabled us to examine various purchasing outcomes indicative of shopping behaviour such as purchasing frequency and dietary quality such as purchasing of fruit and vegetables, UPF and HFSS products. Further, purchasing constitutes a causally more proximal outcome to the NFE exposure investigated than commonly used outcomes such as body weight. The geographical coverage of the study enabled us to assess geographical variation in exposure-outcome associations. Finally, the longitudinal nature of the data enabled us to examine associations at different time points within the same sample of households and individuals.

The study has several limitations. Regarding the spatial context, it is unclear if the neighbourhood as defined in this study is the relevant spatial scale, in terms of both the chosen 1 km network buffer [81–83] as well as the conceptual choice of the NFE [17]. Further spatial error is likely to be introduced by the fact that due to data protection agreements, exact household locations are unknown and NFE exposure is based on population-weighted centroids as address proxies. The distance measures are most likely to be affected by this spatial error. Misclassification of exposure has been shown to bias effect estimates towards the null [28], however, spatial accuracy of area aggregation tends to be better for urban than for rural areas [84]. As most households in this study reside in urban areas, this error might be reduced. Further, if we assume that the spatial error is randomly distributed across the sample, results are internally valid.

With respect to food environment exposure, it has to be noted that even though POI and FHRS are regarded as highly accurate food outlet data sources, they may not have captured all food outlets, especially during periods of rapid change such as between March 2020 (when POI data were collected) and May 2020, with changes including temporary closures of outlets and/or changing from operating as a restaurant to takeaway. Furthermore, in this study we did not address online grocery and takeaway delivery, both of which experienced rapid expansion over the COVID-19 pandemic [11,74]. However, repeating the analysis of take-home purchasing outcomes excluding the purchases made online led to similar results as observed in the main analysis. Hence, we are reasonably confident that online purchasing, which accounts for 11.4% of total purchases, did not bias our analysis. As we restricted our analysis to the seven weeks of the first national lockdown, online purchasing may not have been as relevant as later during the pandemic, when retailers expanded their existing delivery capacities and enabled more households to shop groceries online. Due to limited information, we were not able to restrict OOH purchases to those made from physical premises. Another potential limitation of this study is related to the analytical samples: not all households and individuals who reported purchases in 2019 also reported purchases during lockdown, leaving 57.6% of the 2019 take-home and 38.3% of the 2019 OOH reporters in this analysis. While current samples are similar in terms household and individual characteristics to the full samples (see Tables A and B in S1 File), their reduced sizes result in lower power, potentially missing associations. Equally, the sample of OOH reporters may be underpowered in comparison to take-home reporters to detect associations between OOH purchasing and food environment exposure. Moreover, it is unknown from the household information available whether household composition changed during the pandemic, for example through grown-up children moving back in their parental home. If unaccounted for, such shifts in household composition may bias our estimates of purchasing outcomes. However, the Understanding Society COVID-19 survey reported that household composition remained stable for 95.5% of respondents during lockdown [85]. Finally, using the same parameter specification for every model may not have resulted in the best fit for every association modelled.

## Conclusions

This study investigated associations between NFE exposure and household food and drink purchasing before the COVID-19 pandemic and during the first national lockdown in England, using highly granular, objectively recorded consumer food and drink purchase data. Consistent patterns of exposure-outcome associations were not observed both before the pandemic and during lockdown, when reliance on the local food retail environment was hypothesised to increase. There was some evidence for region-specific effects, highlighting the importance of wider contextual factors in exposure-effect relationships. Future research should consider assessing the impact of the local food environment on those who relied on their NFE during lockdown, while policy makers should focus their efforts on other elements of the food environment which have been more consistently shown to be associated with dietary health.

## Supporting information

S1 File Sample characteristics of those who report during spring 2020 lockdown compared to the full sample in 2019. (PDF)

S2 File Global Moran’s I. (PDF)

S3 File Bivariate associations. (PDF)

S4 File Region-specific analysis. (PDF)

S5 File Sensitivity analysis. (PDF)
