# Supplementary material for: Associations between the neighbourhood food environment and food and drink purchasing in England during lockdown: A repeated cross-sectional analysis
Source: PLoS One. 2024 Jul 17;19(7):e0305295. doi: 10.1371/journal.pone.0305295 (PMC11253942; doi:10.1371/journal.pone.0305295)
Supplement: S1 File — (PDF) [file pone.0305295.s001.pdf]

S1 Sample characteristics of those who report during spring 2020 lockdown compared to the full sample in 2019

Take-home sample

The following table displays descriptive statistics of household characteristics of the full sample and households who did report during lockdown. Differences were formally tested using t tests for numerical and chi-squared tests for categorical variables.

Table A. Comparison of households recording take-home purchases in lockdown and the whole household sample

Mean and standard deviation for numerical variables, n and % for categorical

	Households reporting during lockdown (n=1221)	All households reporting before lockdown (n=2118)
Region ***		
London	527 (43.2%)	1063 (50.2%)
North of England	694 (56.8%)	1055 (49.8%)
Sex of main shopper		
Female	875 (71.7%)	1537 (72.6%)
Male	346 (28.3%)	581 (27.4%)
Age of main shopper (years) ***	54.4 ± 13.4	52.0 ± 14.2
Social grade of main shopper		
AB	270 (22.1%)	498 (23.5%)
C1	522 (43.7%)	907 (42.8%)
C2	204 (16.7%)	331 (15.6%)
D	129 (10.6%)	234 (11.0%)
E	85 (7.0%)	148 (7.0%)
Household size		
1 person	265 (21.7%)	431 (20.3%)
2 persons	465 (38.1%)	765 (36.1%)
3 persons	215 (17.6%)	396 (18.7%)
4 persons	206 (16.9%)	383 (18.1%)
5+ persons	24 (2.0%)	143 (6.8%)
Number of adults		
1	291 (23.8%)	481 (22.7%)
2	656 (53.7%)	1167 (55.1%)
3	176 (14.4%)	296 (14.0%)
4+	98 (8.0%)	174 (8.2%)
Number of children		
0	909 (74.4%)	1501 (70.9%)
1	157 (12.9%)	300 (14.2%)
2	126 (10.3%)	243 (11.5%)
3+	29 (2.4%)	74 (3.5%)

* $p < 0.05$, ** $p < 0.01$, *** $p < 0.001$

Out-of-home sample
The following table displays descriptive statistics of individual characteristics of the full sample in 2019 and of individuals who did report during lockdown. Differences were formally tested using t tests for numerical and chi-squared tests for categorical variables.

Table B. Comparison of individuals recording out-of-home purchases in lockdown and the whole household sample
Mean and standard deviation for numerical variables, n and % for categorical

	Individuals reporting during lockdown (n=171)	All individuals reporting before lockdown (n=447)
Region ***		
London	68 (39.8%)	204 (45.6%)
North of England	103 (60.2%)	243 (54.4%)
Sex of main shopper		
Female	120 (70.2%)	324 (72.5%)
Male	51 (29.8%)	123 (27.5%)
Age of main shopper (years)	50.0 ± 10.9	50.5 ± 12.7
Social grade of main shopper		
AB	33 (19.3%)	107 (23.9%)
C1	82 (48.0%)	210 (47.0%)
C2	27 (15.8%)	67 (15.0%)
D	22 (12.9%)	45 (10.1%)
E	7 (4.1%)	18 (4.0%)
Household size		
1 person	30 (17.5%)	99 (22.1%)
2 persons	72 (42.1%)	165 (36.9%)
3 persons	33 (19.3%)	81 (18.1%)
4 persons	29 (17.0%)	79 (17.7%)
5+ persons	7 (4.1%)	23 (5.1%)
Number of adults		
1	39 (22.8%)	113 (25.3%)
2	95 (55.6%)	248 (55.5%)
3	26 (15.2%)	58 (13.0%)
4+	11 (6.4%)	28 (6.3%)
Number of children		
0	123 (71.9%)	315 (70.5%)
1	24 (14.0%)	66 (14.8%)
2	21 (12.3%)	53 (11.9%)
3+	3 (1.8%)	13 (2.9%)

* $p < 0.05$, ** $p < 0.01$, *** $p < 0.001$

Note that only individuals who recorded purchases and whose characteristics (age, sex etc.) are known. That means that other members of a household than the main reporter who have recorded purchases are not included in this table.
