# Supplementary material for: Associations between the neighbourhood food environment and food and drink purchasing in England during lockdown: A repeated cross-sectional analysis
Source: PLoS One. 2024 Jul 17;19(7):e0305295. doi: 10.1371/journal.pone.0305295 (PMC11253942; doi:10.1371/journal.pone.0305295)
Supplement: S2 File — (PDF) [file pone.0305295.s002.pdf]

S2 Global Moran's I

Table C. Global Moran's I for purchase outcomes

Outcome	Full sample 2019	London 2019	North of England 2019	Full sample 2020	London 2020	North of England 2020
Frequency	0.024	0.037	0.007	0.010	0.037	-0.013
Total energy	0.010	0.011	0.001	0.019	0.030	0.005
Energy from fruit & vegetables	0.003	-0.012	0.001	<-0.001	-0.021	-0.016
Energy from HFSS products	0.008	0.007	-0.001	0.025	0.031	0.012
Energy from UPF	0.016	0.009	0.002	0.041	0.044	0.017
Alcohol volume	0.007	0.010	-0.021	0.022	<-0.001	-0.015
OOH frequency	-0.004	0.060	-0.032	0.001	0.027	-0.016
OOH spend	0.029	0.045	0.011	0.050	0.103	0.027
