# Supplementary material for: Associations between the neighbourhood food environment and food and drink purchasing in England during lockdown: A repeated cross-sectional analysis
Source: PLoS One. 2024 Jul 17;19(7):e0305295. doi: 10.1371/journal.pone.0305295 (PMC11253942; doi:10.1371/journal.pone.0305295)
Supplement: S3 File — (PDF) [file pone.0305295.s003.pdf]

S3 Bivariate associations

Table D. Bivariate associations in take-home sample

2019						
	Purchase occasions	Total energy	Energy from fruit & vegetables	Energy from HFSS products	Energy from UPF	Volume of alcoholic beverages
Region [a]	t=2.43, df=1117.1, p=0.015	t=-0.65, df=1048.4, p=0.513	t=5.49, df=801.5, p<0.001	t=-0.99, df=1110.7, p=0.322	t=-4.18, df=1064.6, p<0.001	t=-5.19, df=1150.7, p<0.001
Population density	rho=0.14, p<0.001	rho=-0.04, p=0.142	rho=0.08, p=0.008	rho<0.01, p=0.867	rho=-0.09, p=0.005	rho=-0.15, p=0.013
Area deprivation	rho=-0.06, p=0.030	rho=-0.03, p=0.369	rho=0.11, p<0.001	rho=-0.06, p=0.039	rho=-0.09, p=0.002	rho=0.07, p=0.013
Age [b]	rho=0.16, p<0.001	rho=0.40, p<0.001	rho=-0.02, p=0.456	rho=0.02, p=0.514	rho=-0.05, p=0.073	rho=0.13, p<0.001
Sex [a]	t=-0.62, df=608.5, p=0.534	t=-1.17, df=593.1, p=0.243	t=1.25, df=575.6, p=0.212	t=0.13, df=539.2, p=0.899	t=1.21, df=553.7, p=0.228	t=-1.45, df=570.8, p=0.149
Children [a]	t=-3.45, df=654.2, p=0.001	t=-15.32, df=946.9, p<0.001	t=-0.39, df=514.5, p=0.699	t=0.42, df=610.1, p=0.672	t=3.46, df=585.9, p<0.001	t=-4.59, df=821.2, p<0.001
Household size [b]	rho=-0.02, p=0.492	rho=-0.43, p<0.001	rho=-0.02, p=0.547	rho=0.04, p=0.198	rho=0.04, p=0.164	rho=-0.06, p=0.025
Social grade [c]	X=6.64, df=8, p=0.575	X=23.43, df=8, p=0.003	X=54.02, df=8, p<0.001	X=20.94, df=8, p=0.007	X=17.99, df=8, p=0.021	X=6.58, df=8, p=0.0.361
Purchase occasions [b]						
Total energy [b]	rho=0.293, p<0.001					
Energy from fruit & vegetables [b]	rho=-0.02, p=0.502	rho=-0.14, p<0.001				
Energy from HFSS products [b]	rho=0.0, p=0.2363	rho=0.15, p<0.001	rho=-0.35, p<0.001			
Energy from UPF [b]	rho=<0.01, p=0.965	rho=-0.02, p=0.508	rho=-0.40, p<0.001	rho=0.32, p<0.001		
Volume of alcoholic beverages [b]	rho=0.09, p=0.002	rho=0.21, p<0.001	rho=-0.07, p=0.009	rho=-0.03, p=0.251	rho=-0.15, p<0.001	
All supermarket density [b]	rho=0.12, p<0.001	rho=-0.04, p=0.147	rho=0.05, p=0.107	rho=0.02, p=0.532	rho=-0.03, p=0.348	rho=-0.13, p<0.001
Chain supermarket density [b]	rho=0.09, p=0.002	rho=-0.01, p=0.673	rho=0.01, p=0.627	rho=0.05, p=0.070	rho=0.01, p=0.761	rho=-0.05, p=0.055
Independent supermarket density [b]	rho=0.11, p<0.001	rho=-0.04, p=0.131	rho=0.07, p=0.017	rho=-0.02, p=0.565	rho=-0.06, p=0.038	rho=-0.15, p<0.001

All supermarket distance [b]	rho=-0.11, p<0.001	rho=0.05, p=0.095	rho=-0.01, p=0.754	rho=-0.02, p=0.438	rho=0.03, p=0.273	rho=0.08, p=0.004
Chain supermarket distance [b]	rho=-0.09, p=0.001	rho=0.04, p=0.206	rho=-0.03, p=0.361	rho=-0.01, p=0.656	rho=0.02, p=0.427	rho=0.06, p=0.032
Independent supermarket distance [b]	rho=-0.12, p<0.001	rho=0.03, p=0.225	rho=-0.05, p=0.099	rho=-0.03, p=0.324	rho=0.03, p=0.299	rho=0.11, p<0.001
OOH outlet density [b]	rho=0.11, p<0.001	rho=-0.02, p=0.423	rho=0.06, p=0.034	rho=-0.01, p=0.968	rho=-0.06, p=0.023	rho=-0.07, p=0.015
Restaurant density [b]	rho=0.11, p<0.001	rho=-0.02, p=0.579	rho=0.09, p=0.002	rho=-0.01, p=0.755	rho=-0.08, p=0.004	rho=-0.06, p=0.027
Takeaway outlet density [b]	rho=0.08, p=0.005	rho=-0.04, p=0.206	rho=0.01, p=0.764	rho<-0.01, p=0.934	rho=-0.03, p=0.253	rho=-0.06, p=0.032
OOH outlet distance [b]	rho=-0.11, p<0.001	rho<0.01, p=0.870	rho=0.01, p=0.742	rho<0.01, p=0.939	rho=0.03, p=0.320	rho=0.07, p=0.010
Restaurant distance [b]	rho=-0.12, p<0.001	rho<-0.01, p=0.961	rho=-0.02, p=0.407	rho<0.01, p=0.946	rho=0.04, p=0.147	rho=0.08, p=0.004
Takeaway outlet distance [b]	rho=-0.10, p<0.001	rho=0.02, p=0.550	rho=-0.01, p=0.618	rho<0.01, p=0.800	rho=0.02, p=0.393	rho=0.07, p=0.023
Composition of food environment [c]	X=13.61, df=8, p=0.093	X=7.89, df=8, p=0.444	X=11.86, df=8, p=0.158	X=10.40, df=8, p=0.238	X=9.62, df=8, p=0.293	X=10.58, df=6, p=0.102

2020

	Purchase occasions	Total energy	Energy from fruit & vegetables	Energy from HFSS products	Energy from UPF	Volume of alcoholic beverages
Region [a]	t=2.03, df=1094.7, p=0.043	t=-0.25, df=1070.2, p=0.804	t=6.55, df=794.3, p<0.001	t=-3.27, df=1078.8, p=0.001	t=-5.82, df=2065.5, p<0.001	t=-6.30, df=1034.8, p<0.001
Population density	rho=0.10, p=0.001	rho=-0.05, p=0.071	rho=0.14, p<0.001	rho=-0.05, p=0.068	rho=-0.13, p<0.001	rho=-0.19, p<0.001
Area deprivation	rho=-0.05, p=0.061	rho=-0.03, p=0.259	rho=0.11, p<0.001	rho=-0.08, p=0.006	rho=-0.08, p=0.004	rho=0.03, p=0.301
Age [b]	rho=0.12, p<0.001	rho=0.22, p<0.001	rho=0.03, p=0.267	rho=-0.09, p=0.001	rho=-0.09, p=0.001	rho=-0.03, p=0.339
Sex [a]	t=-2.64, df=525.7, p=0.009	t=-1.66, df=532.9, p=0.098	t=1.27, df=745.1, p=0.206	t=0.78, df=595.8, p=0.435	t=0.44, df=588.1, p=0.662	t=-1.22, df=554.5, p=0.222
Children [a]	t=-3.90, df=661, p<0.001	t=-13.68, df=910.7, p<0.001	t=-3.60, df=661.5, p<0.001	t=3.30, df=629.5, p=0.001	t=5.24, df=602.8, p<0.001	t=-1.15, df=478.5, p=0.250
Household size [b]	rho=-0.01, p=0.781	rho=-0.38, p<0.001	rho=-0.09, p=0.001	rho=0.06, p=0.044	rho=0.09, p=0.002	rho=0.06, p=0.047
Social grade [c]	X=5.07, df=8, p=0.750	X=13.38, df=8, p=0.100	X=76.02, df=8, p<0.001	X=18.52, df=8, p=0.018	X=33.31, df=8, p<0.001	X=15.04, df=8, p=0.020
Purchase occasions [b]						
Total energy [b]	rho=0.24, p<0.001					

Energy from fruit & vegetables [b]	rho=0.05, p=0.077	rho=-0.09, p=0.001				
Energy from HFSS products [b]	rho=-0.10, p<0.001	rho=0.08, p=0.005	rho=-0.35, p<0.001			
Energy from UPF [b]	rho=-0.07, p=0.018	rho=-0.02, p=0.491	rho=-0.40, p<0.001	rho=0.34, p<0.001		
Volume of alcoholic beverages [b]	rho=0.12, p<0.001	rho=0.23, p<0.001	rho=-0.07, p=0.015	rho=-0.04, p=0.203	rho=-0.10, p=0.001	
All supermarket density [b]	rho=0.08, p=0.004	rho=-0.06, p=0.027	rho=0.07, p=0.013	rho=-0.03, p=0.337	rho=-0.06, p=0.029	rho=-0.15, p<0.001
Chain supermarket density [b]	rho=0.06, p=0.034	rho=-0.05, p=0.083	rho=0.02, p=0.557	rho=0.01, p=0.698	rho=-0.02, p=0.474	rho=-0.09, p=0.001
Independent supermarket density [b]	rho=-0.08, p=0.007	rho=-0.06, p=0.048	rho=0.10, p<0.001	rho=-0.04, p=0.134	rho=-0.09, p=0.003	rho=-0.16, p<0.001
All supermarket distance [b]	rho=-0.10, p<0.001	rho=0.04, p=0.142	rho=-0.05, p=0.080	rho=0.01, p=0.612	rho=0.05, p=0.080	rho=0.07, p=0.009
Chain supermarket distance [b]	rho=-0.10, p<0.001	rho=0.03, p=0.291	rho=-0.02, p=0.389	rho<-0.01, p=0.01	rho=0.02, p=0.435	rho=0.06, p=0.029
Independent supermarket distance [b]	rho=-0.08, p=0.004	rho=0.04, p=0.162	rho=-0.08, p=0.007	rho=0.01, p=0.635	rho=0.07, p=0.016	rho=0.12, p<0.001
OOH outlet density [b]	rho=0.09, p=0.086	rho=-0.04, p=0.152	rho=0.09, p=0.001	rho=-0.06, p=0.042	rho=-0.12, p<0.001	rho=-0.11, p<0.001
Restaurant density [b]	rho=0.10, p<0.001	rho=-0.04, p=0.221	rho=0.14, p<0.001	rho=-0.08, p=0.004	rho=-0.15, p<0.001	rho=-0.11, p<0.001
Takeaway outlet density [b]	rho=0.04, p=0.143	rho=-0.05, p=0.089	rho=0.02, p=0.577	rho=-0.02, p=0.564	rho=-0.07, p=0.023	rho=-0.08, p=0.007
OOH outlet distance [b]	rho=-0.11, p<0.001	rho=0.02, p=0.456	rho=-0.03, p=0.289	rho=0.01, p=0.748	rho=0.03, p=0.286	rho=0.09, p<0.003
Restaurant distance [b]	rho=-0.10, p<0.001	rho=0.02, p=0.574	rho=-0.05, p=0.072	rho=0.05, p=0.106	rho=0.07, p=0.017	rho=0.12, p<0.001
Takeaway outlet distance [b]	rho=-0.09, p=0.001	rho=0.02, p=0.461	rho=-0.03, p=0.255	rho<-0.01, p=0.877	rho=0.04, p=0.184	rho=0.07, p=0.017
Composition of food environment [c]	X=8.51, df=8, p=0.386	X=11.97, df=8, p=0.153	X=12.55, df=8, p=0.128	X=9.94, df=8, p=0.269	X=16.28, df=8, p=0.039	X=5.87, df=6, p=0.438

Results (test statistic/effect size and estimated p-value) of bivariate analyses among the study variables. Superscripts indicate the test used. UPF = ultra-processed food; OOH = out-of-home.

[a] Welch two sample t-test

[b] Spearman rank correlation

[c] Chi square test. Purchase measures were categorised into quantiles to reduce the number of parameters.

**Table E**. Bivariate associations among out-of-home sample		
	OOH occasions 2019	OOH occasions 2020
Region [a]	t=-0.65, df=140.5, p=0.516	t=-1.10, df=167.7, p=0.271
Population density	rho=-0.07, p=0.342	rho=0.04, p=0.646
Area deprivation	rho=-0.06, p=0.401	rho=-0.18, p=0.022
Age [b]	rho=0.13, p=0.083	rho=-0.01, p=0.919
Sex [a]	t=-2.48, df=74.52, p=0.015	t=-2.43, df=68.3, p=0.018
Children [a]	t=-0.11, df=76.04, p=0.912	t=0.54, df=72.2, p=0.590
Household size [b]	rho=-0.10, p=0.186	rho=-0.04, p=0.623
Social grade [c]	X=15.72, df=8, p=0.047	X=5.63, df=8, p=0.689
OOH spend	rho=0.71, p<0.001	rho=0.60, p<0.001
All supermarket density [b]	rho=-0.12, p=0.107	rho=0.03, p=0.629
Chain supermarket density [b]	rho=-0.13, p=0.099	rho=0.01, p=0.922
Independent supermarket density [b]	rho=-0.09, p=0.254	rho=0.05, p=0.531
All supermarket distance [b]	rho=0.07, p=0.372	rho=-0.04, p=0.622
Chain supermarket distance [b]	rho=0.02, p=0.804	rho<0.01, p=0.995
Independent supermarket distance [b]	rho=0.09, p=0.231	rho=-0.04, p=0.600
OOH outlet density [b]	rho=-0.12, p=0.121	rho=0.03, p=0.660
Restaurant density [b]	rho=-0.14, p=0.065	rho=0.05, p=0.530
Takeaway outlet density [b]	rho=-0.09, p=0.264	rho=0.05, p=0.528
OOH outlet distance [b]	rho=0.10, p=0.208	rho=-0.02, p=0.775
Restaurant distance [b]	rho=0.08, p=0.274	rho=-0.08, p=0.310
Takeaway outlet distance [b]	rho=0.08, p=0.287	rho<0.01, p=0.990
Composition of food environment [c]	X=8.97, df=8, p=0.345	X=13.78, df=8, p=0.088

Results (test statistic/effect size and estimated p-value) of bivariate analyses among the study variables. Superscripts indicate the test used. OOH = out-of-home.

[a] Welch two sample t-test

[b] Spearman rank correlation

[c] Chi square test. Purchase measures were categorised into quantiles to reduce the number of parameters

Table F. Associations between area characteristics and food environment exposure in take-home and OOH sample and both years

	**2019**			
	Area deprivation		Population density	
	Take-home sample	OOH sample	Take-home sample	OOH sample
Supermarket density	rho=-0.30, p<0.001	rho=-0.37, p<0.001	rho=0.71, p<0.001	rho=0.79, p<0.001
Supermarket distance	rho=0.20, p<0.001	rho=0.24, p=0.001	rho=-0.56, p<0.001	rho=-0.63, p<0.001
Chain supermarket density	rho=-0.22, p<0.001	rho=-0.31, p<0.001	rho=0.51, p<0.001	rho=0.60, p<0.001
Chain supermarket distance	rho=0.17, p<0.001	rho=0.19, p=0.013	rho=-0.49, p<0.001	rho=-0.53, p<0.001
Independent supermarket density	rho=-0.28, p<0.001	rho=-0.32, p<0.001	rho=0.71, p<0.001	rho=0.77, p<0.001
Independent supermarket distance	rho=0.23, p<0.001	rho=0.25, p=0.001	rho=-0.64, p<0.001	rho=-0.68, p<0.001
OOH outlet density	rho=-0.12, p<0.001	rho=-0.19, p=0.011	rho=0.64, p<0.001	rho=0.71, p<0.001
OOH outlet distance	rho=0.19, p<0.001	rho=0.24, p=0.001	rho=-0.55, p<0.001	rho=-0.58, p<0.001
Restaurant density	rho<0.01, p=0.962	rho=-0.11, p=0.165	rho=0.62, p<0.001	rho=0.68, p<0.001
Restaurant distance	rho=0.08, p=0.004	rho=0.18, p=0.021	rho=-0.62, p<0.001	rho=-0.60, p<0.001
Takeaway outlet density	rho=-0.26, p<0.001	rho=-0.32, p<0.001	rho=0.54, p<0.001	rho=0.61, p<0.001
Takeaway outlet distance	rho=0.21, p<0.001	rho=0.22, p=0.003	rho=-0.54, p<0.001	rho=-0.59, p<0.001
Food environment composition	Kruskal-Wallis chi-squared=73.95, df=2, p<0.001	Kruskal-Wallis chi-squared=23.68, df=2, p<0.001	Kruskal-Wallis chi-squared=209.45, df=2, p<0.001	Kruskal-Wallis chi-squared=43.79, df=2, p<0.001
	**2020**			
	Area deprivation		Population density	
	Take-home sample	OOH sample	Take-home sample	OOH sample
Supermarket density	rho=-0.32, p<0.001	rho=-0.37, p<0.001	rho=0.78, p<0.001	rho=0.75, p<0.001
Supermarket distance	rho=0.19, p<0.001	rho=0.23, p=0.002	rho=-0.63, p<0.001	rho=-0.58, p<0.001
Chain supermarket density	rho=-0.25, p<0.001	rho=-0.34, p<0.001	rho=0.60, p<0.001	rho=0.56, p<0.001

Chain supermarket distance	rho=0.17, p<0.001	rho=0.20, p=0.010	rho=-0.54, p<0.001	rho=-0.50, p<0.001
Independent supermarket density	rho=-0.26, p<0.001	rho=-0.29, p<0.001	rho=0.76, p<0.001	rho=0.74, p<0.001
Independent supermarket distance	rho=0.23, p<0.001	rho=0.24, p=0.001	rho=-0.54, p<0.001	rho=-0.65, p<0.001
OOH outlet density	rho=-0.12, p<0.001	rho=-0.19, p=0.012	rho=0.72, p<0.001	rho=0.69, p<0.001
OOH outlet distance	rho=0.17, p<0.001	rho=0.23, p=0.003	rho=-0.57, p<0.001	rho=-0.55, p<0.001
Restaurant density	rho=0.01, p=0.858	rho=-0.10, p=0.179	rho=0.69, p<0.001	rho=0.67, p<0.001
Restaurant distance	rho=0.08, p=0.004	rho=0.17, p=0.022	rho=-0.60, p<0.001	rho=-0.61, p<0.001
Takeaway outlet density	rho=-0.27, p<0.001	rho=-0.32, p<0.001	rho=0.62, p<0.001	rho=0.59, p<0.001
Takeaway outlet distance	rho=0.21, p<0.001	rho=0.20, p=0.008	rho=-0.59, p<0.001	rho=-0.54, p<0.001
Food environment composition	Kruskal-Wallis chi-squared=77.16, df=2, p<0.001	Kruskal-Wallis chi-squared=27.51, df=2, p<0.001	Kruskal-Wallis chi-squared=209.57, df=2, p<0.001	Kruskal-Wallis chi-squared=44.82, df=2, p<0.001

Spearman rank correlation for all associations except those concerning the food environment composition, which were tested using Kruskal-Wallis test.

Table G. Associations between region and food environment exposure

Exposure measure	Take-home sample	Out-of-home sample
	**2019**	
Density of all supermarkets	t=17.73, df=708.7, p<0.001	t=7.91, df=99.59, p<0.001
Distance to nearest supermarket (any)	t=-10.47, df=855.3, p<0.001	t=-5.99, df=130.7, p<0.001
Density of chain supermarkets	t=8.40, df=1087.8, p<0.001	t=4.58, df=142.7, p<0.001
Distance to nearest chain supermarket	t=-9.81, df=839.4, p<0.001	t=-4.94, df=119.9, p<0.001
Density of independent supermarkets	t=18.72, df=632.1, p<0.001	t=7.86, df=86.93, p<0.001
Distance to nearest independent supermarket	t=-14.9, df=818.6, p<0.001	t=-6.59, df=126.8, p<0.001
Density of OOH outlets	t=13.18, df=886.6, p<0.001	t=6.42, df=103.1, p<0.001
Distance to nearest OOH outlet	t=-10.96, df=832.8, p<0.001	t=-4.82, df=117.7, p<0.001
Density of restaurants	t=14.25, df=824.0, p<0.001	t=6.69, df=81.9, p<0.001
Distance to nearest restaurant	t=-15.21, df=809.6, p<0.001	t=-6.33, df=117.2, p<0.001
Density of takeaway outlets	t=6.83, df=1094.9, p<0.001	t=3.39, df=155.6, p=0.001
Distance to nearest takeaway outlets	t=-10.67, df=828.4=, p<0.001	t=-4.68, df=118.6, p<0.001
Food environment composition	X=85.3, df=2, p<0.001	X=17.47, df=2, p<0.001
	**2020**	
Density of all supermarkets	t=17.22, df=773.8, p<0.001	t=11.94, df=101.2, p<0.001
Distance to nearest supermarket (any)	t=-11.20, df=876.9, p<0.001	t=-6.03, df=130.4, p<0.001
Density of chain supermarkets	t=7.75, df=1117.1, p<0.001	t=4.29, df=148.3, p<0.001
Distance to nearest chain supermarket	t=-9.68, df=842.4, p<0.001	t=-5.00, df=119.7, p<0.001
Density of independent supermarkets	t=18.42, df=642.5, p<0.001	t=7.61, df=88.1, p<0.001
Distance to nearest independent supermarket	t=-15.91, df=829.9, p<0.001	t=-7.34, df=120.32, p<0.001
Density of OOH outlets	t=13.16, df=888.1, p<0.001	t=6.46, df=105.8, p<0.001
Distance to nearest OOH outlet	t=-10.78, df=831.9, p<0.001	t=-4.64, df=117.8, p<0.001
Density of restaurants	t=14.19, df=823.4, p<0.001	t=6.78, df=82.5, p<0.001
Distance to nearest restaurant	t=-14.81, df=809.4, p<0.001	t=-6.2, df=117.2, p<0.001
Density of takeaway outlets	t=6.74, df=1106.7, p=0.001	t=3.25, df=162.7, p=0.001
Distance to nearest takeaway outlets	t=-10.53, df=820.6=, p<0.001	t=-4.57, df=119.5=, p<0.001
Food environment composition	X=95.47, df=2, p<0.001	X=20.70, df=2, p<0.001
