# Supplementary material for: Associations between the neighbourhood food environment and food and drink purchasing in England during lockdown: A repeated cross-sectional analysis
Source: PLoS One. 2024 Jul 17;19(7):e0305295. doi: 10.1371/journal.pone.0305295 (PMC11253942; doi:10.1371/journal.pone.0305295)
Supplement: S4 File — (PDF) [file pone.0305295.s004.pdf]

S4 Region-specific analysis

Table H. Region-specific parameter estimates and 95% CI of take-home purchase outcomes associated with food environment exposures, 2019 and 2020

		Adjusted Estimates																	
		Frequency			Total energy			Energy from FV			Energy from HFSS products			Energy from UPF			Alcohol volume		
Exposure	Region	IR	95% CI	*p* value	IR	95% CI	*p* value	IR	95% CI	P value	IR	95% CI	*p* value	IR	95% CI	*p* value	IR	95% CI	*p* value
	2019																		
Density of chain supermarkets	London	0.993	0.970, 1.017	0.767	1.005	0.985, 1.026	0.694	1.013	0.979, 1.047	0.992	0.996	0.986, 1.007	0.824	1.002	0.990, 1.015	0.895	0.981	0.834, 1.154	0.973
	NE	1.001	0.985, 1.018	0.882	0.997	0.983, 1.011	0.720	0.999	0.977, 1.022	0.961	1.004	0.997, 1.011	0.990	1.005	0.997, 1.013	0.802	0.963	0.861, 1.077	0.904
Distance to chain supermarkets	London	1.075*	1.009, 1.145	0.194	1.046	0.991, 1.104	0.281	1.000	0.916, 1.092	0.992	1.001	0.974, 1.030	0.926	1.005	0.973, 1.038	0.895	1.074	0.673, 1.713	0.973
	NE	1.027*	0.994, 1.061	0.843	1.025	0.997, 1.054	0.208	1.008	0.963, 1.055	0.961	0.996	0.982, 1.011	0.990	0.997	0.980, 1.014	0.949	1.045	0.823, 1.327	0.904
Density of independent supermarkets	London	1.001	0.991, 1.010	0.912	0.999	0.991, 1.006	0.718	0.998	0.986, 1.011	0.992	0.998	0.994, 1.002	0.824	1.001	0.996, 1.005	0.895	0.984	0.926, 1.045	0.973
	NE	0.998	0.988, 1.008	0.882	0.996	0.988, 1.005	0.539	1.001	0.987, 1.016	0.961	1.000	0.995, 1.004	0.990	1.000	0.995, 1.005	0.972	0.936	0.873, 1.004	0.518
Distance to independent supermarkets	London	0.995	0.930, 1.065	0.912	1.075	1.016, 1.138	0.051	1.003	0.913, 1.101	0.992	1.005	0.975, 1.035	0.867	1.011	0.977, 1.046	0.895	0.972	0.598, 1.580	0.973
	NE	0.993	0.959, 0.681	0.882	1.040	1.010, 1.071	0.033	1.006	0.959, 1.055	0.961	1.000	0.985, 1.016	0.990	1.001	0.984, 1.019	0.972	0.985	0.767, 1.265	0.904
Density of OOH outlets	London	1.002	0.999, 1.006	0.558	1.001	0.998, 1.004	0.687	1.001	0.996, 1.006	0.992	0.999	0.998, 1.001	0.824	1.001	0.999, 1.002	0.895	1.000	0.977, 1.024	0.973
	NE	1.002	0.999, 1.004	0.882	0.999	0.997, 1.001	0.539	1.000	0.996, 1.004	0.971	1.000	0.999, 1.001	0.990	1.000	0.998, 1.001	0.949	0.995	0.977, 1.014	0.904
Distance to OOH outlets	London	1.048	0.968, 1.134	0.558	1.026	0.959, 1.097	0.687	1.016	0.911, 1.133	0.992	1.009	0.974, 1.045	0.841	0.997	0.958, 1.038	0.895	1.329	0.775, 2.2278	0.973
	NE	1.012	0.972, 1.054	0.882	1.016	0.982, 1.052	0.539	1.019	0.964, 1.078	0.961	1.000	0.982, 1.018	0.990	0.991	0.971, 1.012	0.802	1.154	0.876, 1.519	0.904
Composition of food environments																			
More OOH	London	0.958	0.848, 1.083	0.767	1.048	0.945, 1.162	0.687	1.055	0.890, 1.249	0.992	0.976	0.925, 1.031	0.824	0.993	0.933, 1.057	0.895	1.044	0.472, 2.313	0.973
	NE	0.987	0.915, 1.064	0.882	1.012	0.949, 1.078	0.720	1.022	0.921, 1.135	0.961	0.979	0.947, 1.012	0.990	0.978	0.941, 1.016	0.802	1.071	0.654, 1.753	0.904

No outlets	London	1.141	0.898, 1.450	0.558	1.547*	1.261, 1.897	<0.001	0.982	0.703, 1.372	0.992	1.069	0.960, 1.189	0.824	1.022	0.904, 1.156	0.895	0.782	0.159, 3.855	0.973
	NE	0.990	0.865, 1.133	0.882	1.224*	1.092, 1.373	0.004	1.077	0.893, 1.299	0.961	1.000	0.942, 1.062	0.990	1.011	0.928, 1.101	0.802	1.085	0.444, 2.651	0.904
	2020																		
Density of chain supermarkets	London	0.997	0.974, 1.021	0.868	0.979	0.960, 0.999	0.222	1.001	0.971, 1.031	0.958	1.004	0.994, 1.014	0.816	0.999	0.986, 1.012	0.924	0.952	0.832, 1.090	0.954
	NE	0.995	0.980, 1.011	0.637	0.982	0.969, 0.994	0.042	0.992	0.973, 1.012	0.930	1.003	0.997, 1.010	0.910	1.000	0.992, 1.009	0.931	0.956	0.869, 1.052	0.931
Distance to chain supermarkets	London	1.013	0.953, 1.078	0.868	1.046	0.992, 1.103	0.249	0.973	0.899, 1.054	0.958	0.994	0.968, 1.020	0.834	1.003	0.970, 1.038	0.924	1.039	0.698, 1.545	0.954
	NE	0.994	0.963, 1.027	0.726	1.021	0.994, 1.049	0.208	0.997	0.957, 1.039	0.930	0.995	0.982, 1.009	0.983	0.997	0.980, 1.015	0.931	0.999	0.814, 1.224	0.989
Density of independent supermarkets	London	0.996	0.987, 1.005	0.718	0.996	0.988, 1.004	0.449	1.001	0.989, 1.013	0.958	0.998	0.994, 1.002	0.816	0.997	0.992, 1.002	0.919	1.002	0.950, 1.056	0.954
	NE	0.991	0.981, 1.001	0.514	0.992	0.984, 1.000	0.128	0.999	0.987, 1.012	0.930	1.000	0.995, 1.004	0.983	0.997	0.991, 1.002	0.648	0.980	0.922, 1.042	0.931
Distance to independent supermarkets	London	0.950	0.886, 1.018	0.469	1.059	0.999, 1.124	0.222	1.048	0.958, 1.146	0.958	1.004	0.975, 1.034	0.854	1.011	0.973, 1.051	0.919	0.809	0.520, 1.258	0.954
	NE	0.973	0.939, 1.008	0.514	1.030	0.999, 1.062	0.128	1.029	0.983, 1.077	0.882	1.000	0.985, 1.015	0.983	1.002	0.983, 1.022	0.931	0.894	0.713, 1.122	0.931
Density of OOH outlets	London	1.003	0.999, 1.006	0.469	0.999	0.996, 1.002	0.696	0.999	0.994, 1.003	0.958	1.000	0.999, 1.002	0.816	0.999	0.998, 1.001	0.919	0.996	0.976, 1.017	0.954
	NE	1.001	0.999, 1.004	0.637	0.997	0.995, 1.000	0.071	0.999	0.996, 1.002	0.930	1.000	0.999, 1.001	0.983	0.998	0.997, 0.999	0.033	0.999	0.983, 1.016	0.989
Distance to OOH outlets	London	0.981	0.906, 1.062	0.868	1.034	0.967, 1.106	0.449	0.989	0.893, 1.094	0.958	1.003	0.970, 1.037	0.854	0.998	0.955, 1.042	0.924	1.201	0.732, 1.971	0.954
	NE	0.982	0.943, 1.023	0.637	1.014	0.980, 1.050	0.479	1.010	0.958, 1.064	0.930	0.998	0.981, 1.015	0.983	0.993	0.971, 1.016	0.925	1.074	0.833, 1.384	0.931
Composition of food environments																			
More OOH	London	0.915	0.805, 1.040	0.469	1.008	0.902, 1.126	0.889	0.988	0.836, 1.167	0.958	1.041	0.986, 1.100	0.591	0.976	0.909, 1.048	0.919	1.058	0.488, 2.294	0.954
	NE	0.975	0.903, 1.053	0.637	0.994	0.930, 1.062	0.850	0.993	0.899, 1.098	0.930	1.017	0.984, 1.051	0.910	0.975	0.934, 1.017	0.648	1.169	0.729, 1.875	0.931
No outlets	London	0.980	0.772, 1.244	0.868	1.164	0.948, 1.431	0.295	1.092	0.800, 1.491	0.958	1.111	1.003, 1.230	0.342	1.119	0.980, 1.278	0.775	0.554	0.126, 2.433	0.954
	NE	0.956	0.821, 1.144	0.637	1.077	0.960, 1.209	0.275	1.127	0.947, 1.341	0.882	1.041	0.983, 1.102	0.910	1.021	0.948, 1.100	0.925	0.924	0.404, 2.114	0.989

95% CI = 95% confidence interval; FV = Fruit & vegetables; IR = Incidence Rate; NE = North of England; OOH = out of home.
* Effect interaction was detected (p<0.005); see Table S12 for interaction parameters.
Effect estimates of density measures refer to a change in incidence rate in response to an increase of 1 m/km^2. Effect estimates of distance measures refer to a change in incidence rate in response to an increase of 500 m. The reference category for the composition of food environments is neighbourhoods with more supermarkets.
All models were adjusted for age, sex and NRS social grade of the main shopper, number of children and adults in the household, region, area deprivation and population density, and interactions between region and NRS social grade, area deprivation, and population density. *P* values were adjusted for multiple testing using the Benjamini-Hochberg method.

Table I. Region-specific parameter estimates and 95% CI of OOH purchasing associated with food environment exposures, 2019 and 2020

		Adjusted Estimates		
Exposure	Region	IR	95% CI	*p* value
		2019		
Density of all supermarkets	London	0.969	0.930, 1.009	0.192
	NE	0.969	0.940, 1.000	0.178
Distance to any supermarket	London	0.899	0.590, 1.370	0.619
	NE	0.905	0.728, 1.127	0.372
Density of restaurants	London	0.978	0.957, 1.000	0.133
	NE	0.984	0.964, 1.004	0.178
Distance to restaurants	London	0.654	0.438, 0.977	0.133
	NE	0.799	0.651, 0.981	0.178
Density of takeaway outlets	London	0.979	0.922, 1.039	0.543
	NE	0.984	0.950, 1.019	0.372
Distance to takeaway outlets	London	0.781	0.561, 1.088	0.192
	NE	0.867	0.732, 1.026	0.178
Composition of food environments				
More OOH	London	0.570	0.328, 0.991	0.133
	NE	0.769	0.546, 1.083	0.178
No outlets	London	0.637	0.379, 1.073	0.180
	NE	0.637	0.379, 1.073	0.178
		2020		
Density of all supermarkets	London	0.978	0.931, 1.027	0.732
	NE	0.975	0.940, 1.011	0.697
Distance to any supermarket	London	0.860	0.510, 1.450	0.858
	NE	0.889	0.678, 1.165	0.697
Density of restaurants	London	0.984	0.958, 1.011	0.685
	NE	0.996	0.973, 1.020	0.752
Distance to restaurants	London	0.732	0.450, 1.192	0.685
	NE	0.855	0.667, 1.095	0.697
Density of takeaway outlets	London	0.989	0.915, 1.068	0.881
	NE	0.991	0.948, 1.035	0.752
Distance to takeaway outlets	London	0.785	0.517, 1.192	0.685
	NE	0.887	0.718, 1.097	0.697
Composition of food environments				
More OOH	London	1.008	0.446, 2.280	0.984
	NE	1.209	0.750, 1.949	0.697
No outlets	London	0.860	0.453, 1.632	0.858
	NE	0.860	0.453, 1.632	0.742

95% CI = 95% confidence interval; OOH = out of home; IR = Incidence Rate; NE = North of England. Effect estimates of density measures refer to a change in incidence rate in response to an increase of 1 m/km^2. Effect estimates of distance measures refer to a change in incidence rate in response to an increase of 500 m. The reference category for the composition of food environments is neighbourhoods with more supermarkets.
All models were adjusted for age, sex NRS social grade, number of children and adults in the household, region, area deprivation and population density, and interactions between region and NRS social grade, area deprivation, and population density. *P* values were adjusted for multiple testing using the Benjamini-Hochberg method. Note that no interaction terms could be calculated for ‘no outlets’, because no individuals in the OOH sample in London lived in neighbourhoods without any food outlets.

Table J. Parameter estimates and 95% CI of interaction terms between food environment exposure and region on the effect of take-home purchase outcomes

	Frequency			Total energy			Energy from fruit & vegetables			Calories from HFSS products			Calories from UPF			Alcohol volume		
Exposure	IR	95% CI	*p* value	IR	95% CI	*p* value	IR	95% CI	*p* value	IR	95% CI	*p* value	IR	95% CI	*p* value	IR	95% CI	*p* value
2019																		
Density of chain supermarkets	1.017	0.984, 1.050	0.629	0.983	0.957, 1.010	0.357	0.974	0.931, 1.018	0.919	1.015	1.000, 1.029	0.178	1.005	0.989, 1.022	0.639	0.962	0.769, 1.204	0.922
Distance to chain supermarkets	0.913	0.856, 0.974	0.049	0.962	0.910, 1.017	0.335	1.015	0.927, 1.112	0.919	0.990	0.962, 1.019	0.673	0.984	0.952, 1.018	0.639	0.947	0.587, 1.530	0.836
Density of independent supermarkets	0.995	0.975, 1.016	0.723	0.995	0.978, 1.013	0.612	1.006	0.978, 1.035	0.919	1.003	0.994, 1.012	0.673	0.999	0.988, 1.009	0.639	0.906	0.788, 1.041	0.836
Distance to independent supermarkets	0.995	0.929, 1.067	0.894	0.936	0.883, 0.992	0.106	1.007	0.915, 1.107	0.919	0.992	0.962, 1.022	0.673	0.980	0.946, 1.015	0.639	1.027	0.624, 1.691	0.922
Density of OOH outlets	0.998	0.993, 1.003	0.632	0.996	0.992, 1.000	0.205	0.998	0.991, 1.005	0.919	1.001	0.999, 1.004	0.581	0.998	0.996, 1.001	0.639	0.990	0.955, 1.026	0.922
Distance to OOH outlets	0.934	0.861, 1.013	0.260	0.982	0.916, 1.052	0.612	1.006	0.899, 1.125	0.919	0.983	0.949, 1.019	0.673	0.988	0.948, 1.029	0.639	0.753	0.434, 1.306	0.836
Food environment composition																		
More OOH outlets	1.060	0.912, 1.231	0.632	0.932	0.821, 1.058	0.368	0.940	0.763, 1.157	0.919	1.005	0.941, 1.074	0.877	0.970	0.899, 1.047	0.639	1.051	0.386, 2.861	0.922
No outlets	0.752	0.575, 0.985	0.154	0.627	0.499, 0.788	<0.001	1.202	0.827, 1.748	0.919	0.876	0.777, 0.988	0.178	0.898	0.783, 1.031	0.639	1.927	0.319, 11.627	0.922
2020																		
Density of chain supermarkets	0.996	0.966, 1.028	0.934	1.005	0.979, 1.032	0.697	0.983	0.945, 1.023	0.874	0.998	0.985, 1.011	0.808	1.003	0.986, 1.020	0.946	1.008	0.833, 1.220	0.932
Distance to chain supermarkets	0.963	0.904, 1.027	0.497	0.953	0.902, 1.006	0.256	1.050	0.967, 1.140	0.874	1.004	0.977, 1.031	0.808	0.988	0.954, 1.023	0.946	0.925	0.614, 1.391	0.822

Density of independent supermarkets	0.990	0.970, 1.010	0.513	0.992	0.975, 1.009	0.453	0.997	0.972, 1.023	0.917	1.003	0.995, 1.012	0.781	0.999	0.988, 1.010	0.946	0.958	0.847, 1.083	0.822
Distance to independent supermarkets	1.048	0.976, 1.126	0.497	0.945	0.890, 1.004	0.256	0.964	0.880, 1.057	0.874	0.992	0.962, 1.022	0.781	0.982	0.944, 1.021	0.946	1.223	0.778, 1.922	0.822
Density of OOH outlets	0.997	0.992, 1.002	0.497	0.996	0.992, 1.001	0.256	1.000	0.994, 1.007	0.917	0.998	0.996, 1.001	0.427	0.997	0.994, 1.000	0.147	1.006	0.973, 1.040	0.822
Distance to OOH outlets	1.003	0.924, 1.088	0.946	0.962	0.898, 1.031	0.442	1.043	0.940, 1.158	0.874	0.990	0.957, 1.025	0.781	0.991	0.947, 1.036	0.946	0.799	0.481, 1.327	0.822
Food environment composition																		
More OOH outlets	1.135	0.973, 1.324	0.497	0.972	0.851, 1.109	0.697	1.012	0.828, 1.236	0.917	0.954	0.894, 1.019	0.427	0.997	0.915, 1.086	0.946	1.222	0.479, 3.119	0.822
No outlets	0.952	0.728, 1.245	0.934	0.856	0.680, 1.078	0.372	1.065	0.752, 1.509	0.917	0.878	0.783, 0.984	0.201	0.833	0.718, 0.966	0.127	2.787	0.528, 14.713	0.822

95% CI = 95% confidence interval; IR = Incidence Rate; OOH = out of home. London is coded as the baseline region.
All models are adjusted for age, sex and NRS social grade of the main shopper, number of children and adults in the household, region, area deprivation and population density, and interactions between region and NRS social grade, area deprivation, and population density. *p* values were adjusted for multiple testing using the Benjamini-Hochberg method.

Table K. Parameter estimates and 95% CI of interaction terms between food environment exposure and region on the effect of OOH purchasing

Exposure	IR	95% CI	*p* value
	2019		
Density of all supermarkets	1.002	0.942, 1.066	0.955
Distance to any supermarket	1.015	0.655, 1.572	0.955
Density of restaurants	1.011	0.971, 1.053	0.955
Distance to restaurants	1.493	0.993, 2.245	0.263
Density of takeaway outlets	1.011	0.943, 1.084	0.955
Distance to takeaway outlets	1.232	0.879, 1.727	0.529
Composition of food environments			
More OOH	1.823	0.941, 3.531	0.263
No outlets	NA	NA	NA
	2020		
Density of all supermarkets	0.995	0.924, 1.071	0.930
Distance to any supermarket	1.068	0.621, 1.836	0.930
Density of restaurants	1.025	0.978, 1.074	0.725
Distance to restaurants	1.362	0.831, 2.232	0.725
Density of takeaway outlets	1.004	0.920, 1.096	0.930
Distance to takeaway outlets	1.276	0.834, 1.952	0.725
Composition of food environments			
More OOH	1.438	0.570, 3.627	0.773
No outlets	NA	NA	NA

95% CI = 95% confidence interval; OOH = out of home; IR = Incidence Rate. London is coded as the baseline region.
All models are adjusted for age, sex NRS social grade, number of children and adults in the household, region, area deprivation and population density, and interactions between region and NRS social grade, area deprivation, and population density. *p* values were adjusted for multiple testing using the Benjamini-Hochberg method. Note that no interaction terms could be calculated for 'no outlets', because no individuals in the OOH sample in London lived in neighbourhoods without any food outlets.
