# Supplementary material for: Associations between the neighbourhood food environment and food and drink purchasing in England during lockdown: A repeated cross-sectional analysis
Source: PLoS One. 2024 Jul 17;19(7):e0305295. doi: 10.1371/journal.pone.0305295 (PMC11253942; doi:10.1371/journal.pone.0305295)
Supplement: S5 File — (PDF) [file pone.0305295.s005.pdf]

S5 Sensitivity analysis

1. Buffer size

Table L. Sensitivity analysis of varying buffer sizes applied to selected models

Model (exposure & outcome)	Year	1 km buffer			0.5 km buffer			2 km buffer			5 km buffer		
		IR	95% CI	*p* value	IR	95% CI	*p* value	IR	95% CI	*p* value	IR	95% CI	*p* value
OOH outlet density & frequency	2019	1.002	0.999, 1.004	0.220	1.001	1.000, 1.003	0.058	1.003	0.999, 1.007	0.095	1.000	0.994, 1.005	0.949
	2020	1.001	0.999, 1.004	0.274	1.001	1.000, 1.003	0.058	1.004	1.001, 1.007	0.020	1.003	0.998, 1.008	0.241
Independent supermarket density & total energy	2019	0.998	0.991, 1.005	0.519	1.001	0.997, 1.005	0.605	0.995	0.982, 1.008	0.444	0.988	0.966, 1.010	0.280
	2020	0.995	0.988, 1.001	0.120	1.001	0.998, 1.005	0.521	0.988	0.976, 1.001	0.076	0.988	0.966, 1.010	0.286
Chain supermarket density & energy from fruit and vegetables	2019	0.997	0.976, 1.010	0.814	1.002	0.991, 1.013	0.721	0.947	0.911, 0.985	0.006	0.949	0.880, 1.023	0.174
	2020	0.991	0.971, 1.010	0.353	0.995	0.985, 1.004	0.283	0.952	0.928, 0.988	0.009	0.945	0.879, 1.014	0.118
Independent supermarket density & energy from HFSS products	2019	0.999	0.995, 1.002	0.485	1.000	0.998, 1.002	0.971	0.999	0.992, 1.006	0.865	0.995	0.984, 1.007	0.447
	2020	0.999	0.995, 1.002	0.460	1.000	0.998, 1.001	0.726	0.999	0.993, 1.005	0.768	0.999	0.988, 1.010	0.814
OOH outlet density & energy from UPF	2019	1.000	0.998, 1.001	0.775	1.000	0.999, 1.000	0.463	1.000	0.998, 1.002	0.895	1.001	0.998, 1.003	0.647
	2020	0.998	0.997, 0.999	0.007	0.999	0.999, 1.000	0.064	0.999	0.997, 1.001	0.542	1.001	0.998, 1.004	0.512
Chain supermarket density & alcohol volume	2019	0.962	0.860, 1.076	0.495	0.981	0.929, 1.036	0.491	0.961	0.781, 1.182	0.706	1.006	0.665, 1.523	0.976
	2020	0.956	0.869, 1.052	0.359	0.982	0.937, 1.030	0.452	1.034	0.854, 1.253	0.731	1.225	0.853, 1.760	0.272

Restaurant density & OOH purchasing	2019	0.982	0.964, 1.000	0.053	1.002	0.993, 1.012	0.594	0.968	0.943, 0.995	0.020	0.971	0.939, 1.004	0.087
	2020	0.992	0.970, 1.014	0.451	1.000	0.989, 1.011	0.952	0.983	0.956, 1.011	0.225	0.990	0.954, 1.029	0.619

95% CI = 95% confidence interval; HFSS = high in fat, salt and sugar; IR = Incidence Rate; OOH = out of home; UPF = ultra-processed foods. Effect estimates of density measures refer to a change in incidence rate in response to an increase of 1 m/km^2. All models are adjusted for age, sex NRS social grade, number of children and adults in the household, region, area deprivation and population density, and interactions between region and NRS social grade, area deprivation, and population density. Note that *p* values have not been adjusted for multiple testing.

2. Varying aggregations of supermarket definitions

Table M. Sensitivity analysis of effects of varying aggregations of supermarket definitions on take-home purchase outcomes

		Adjusted Estimates																	
		Frequency			Total Calories			Calories from fruit & vegetables			Calories from HFSS products			Calories from UPF			Alcohol volume		
Exposure	Year	IR	95% CI	*p* value	IR	95% CI	*p* value	IR	95% CI	*p* value	IR	95% CI	*p* value	IR	95% CI	*p* value	IR	95% CI	*p* value
A density	2019	1.002	0.978, 1.026	0.879	0.991	0.972, 1.012	0.409	1.011	0.978, 1.044	0.535	1.004	0.993, 1.014	0.515	1.003	0.990, 1.015	0.680	0.980	0.838, 1.145	0.795
	2020	0.990	0.965, 1.016	0.445	0.965	0.945, 0.986	0.001	1.010	0.977, 1.043	0.563	0.997	0.987, 1.008	0.608	0.998	0.984, 1.012	0.747	0.955	0.817, 1.117	0.567
A distance	2019	0.991	0.978, 1.005	0.197	1.007	0.996, 1.019	0.217	1.012	0.994, 1.031	0.194	0.993	0.987, 0.998	0.014	0.991	0.984, 0.998	0.010	1.015	0.931, 1.108	0.733
	2020	0.988	0.975, 1.002	0.082	1.003	0.992, 1.014	0.603	1.020	1.002, 1.037	0.025	0.997	0.991, 1.003	0.293	0.991	0.984, 0.999	0.022	0.980	0.904, 1.062	0.621
B density	2019	1.003	0.978, 1.029	0.791	0.999	0.978, 1.021	0.954	0.981	0.948, 1.016	0.295	1.008	0.997, 1.019	0.170	1.011	0.998, 1.024	0.109	0.923	0.764, 1.114	0.403
	2020	0.997	0.975, 1.020	0.828	0.987	0.969, 1.007	0.195	0.973	0.945, 1.001	0.061	1.009	0.999, 1.019	0.069	1.003	0.991, 1.016	0.621	0.935	0.805, 1.085	0.373
B distance	2019	0.985	0.972, 0.998	0.026	1.006	0.996, 1.017	0.256	1.015	0.998, 1.032	0.094	0.994	0.989, 0.999	0.030	0.991	0.985, 0.997	0.005	1.004	0.940, 1.072	0.912
	2020	0.976	0.962, 0.991	0.001	1.000	0.989, 1.012	0.953	1.017	0.999, 1.036	0.059	0.998	0.992, 1.004	0.443	0.993	0.986, 1.001	0.095	0.974	0.984, 1.062	0.553
C density	2019	1.000	0.991, 1.008	0.908	0.998	0.991, 1.005	0.519	1.000	0.988, 1.011	0.951	0.999	0.995, 1.002	0.485	1.000	0.996, 1.005	0.883	0.968	0.916, 1.022	0.238
	2020	0.994	0.986, 1.002	0.123	0.995	0.988, 1.001	0.120	1.000	0.990, 1.011	0.974	0.999	0.995, 1.002	0.460	0.997	0.933, 1.002	0.198	0.994	0.948, 1.042	0.800
C distance	2019	0.991	0.976, 1.006	0.219	1.010	0.97, 1.023	0.123	1.009	0.988, 1.030	0.401	0.997	0.990, 1.003	0.314	0.992	0.985, 0.999	0.035	0.997	0.892, 1.113	0.953
	2020	0.993	0.978, 1.009	0.412	1.005	0.992, 1.018	0.460	1.012	0.992, 1.033	0.225	0.996	0.990, 1.003	0.283	0.994	0.985, 1.002	0.155	0.980	0.891, 1.078	0.680
Chains density	2019	1.002	0.986, 1.018	0.789	0.996	0.983, 1.010	0.556	0.997	0.976, 1.020	0.814	1.005	0.998, 1.012	0.194	1.005	0.997, 1.014	0.200	0.962	0.860, 1.076	0.495
	2020	0.995	0.980, 1.009	0.537	0.982	0.969, 0.995	0.006	0.991	0.971, 1.010	0.353	1.003	0.997, 1.010	0.355	0.992	0.983, 1.000	0.051	0.956	0.869, 1.052	0.359

Chains distance	2019	0.987	0.972, 1.003	0.108	1.008	0.995, 1.021	0.250	1.015	0.993, 1.036	0.177	0.992	0.985, 0.999	0.022	0.990	0.982, 0.998	0.012	1.020	0.921, 1.130	0.701
	2020	0.978	0.963, 0.994	0.006	1.000	0.987, 1.013	0.993	1.018	0.999, 1.038	0.068	0.997	0.991, 1.004	0.389	0.992	0.983, 1.000	0.051	0.965	0.879, 1.058	0.445
All density	2019	1.000	0.993, 1.007	0.987	0.998	0.992, 1.003	0.453	0.999	0.990, 1.008	0.883	1.000	0.997, 1.003	0.983	1.001	0.998. 1.004	0.517	0.974	0.932, 1.017	0.229
	2020	0.995	0.989, 1.001	0.130	0.993	0.988, 0.999	0.018	0.998	0.990, 1.007	0.712	1.000	0.997, 1.002	0.849	0.998	0.995, 1.002	0.341	0.989	0.953, 1.027	0.576
All distance	2019	0.982	0.963, 1.001	0.070	1.010	0.994, 1.027	0.224	1.019	0.992, 1.046	0.165	0.991	0.982, 0.999	0.031	0.989	0.979, 0.999	0.024	0.993	0.847, 1.164	0.928
	2020	0.973	0.953, 0.994	0.010	1.001	0.984, 1.018	0.907	1.029	1.003, 1.056	0.030	0.995	0.987, 1.004	0.276	0.990	0.979, 1.001	0.070	0.941	0.824, 1.074	0.364

A = big chain supermarkets; B = small chain supermarkets & convenience symbol groups; C = independent supermarkets; Chains = A & B; all = A, B & C; HFSS = high in fat, salt and sugar; OOH = out-of-home; UPF = ultra-processed foods.

Effect estimates of density measures refer to a change in incidence rate in response to an increase of 1 m/km^2. Effect estimates of distance measures refer to a change in incidence rate in response to an increase of 500 m. The reference category for the composition of food environments is neighbourhoods with more supermarkets.

All models are adjusted for age, sex and NRS social grade of the main shopper, number of children and adults in the household, region, area deprivation and population density, and interactions between region and NRS social grade, area deprivation, and population density. Note that *p* values have not been adjusted for multiple testing.

Table N. Sensitivity analysis of effects of varying aggregations of supermarket definitions on OOH purchasing

Exposure	Year	IR	95% CI	*p* value
A density	2019	0.836	0.736, 0.949	0.006
	2020	0.957	0.815, 1.123	0.590
A distance	2019	0.992	0.940, 1.048	0.783
	2020	1.022	0.957, 1.091	0.515
B density	2019	0.991	0.890, 1.103	0.863
	2020	0.972	0.866, 1.092	0.632
B distance	2019	0.965	0.903, 1.031	0.292
	2020	0.975	0.896, 1.061	0.556
C density	2019	0.970	0.935, 1.007	0.110
	2020	0.973	0.931, 1.016	0.212
C distance	2019	0.977	0.904, 1.056	0.553
	2020	1.029	0.938, 1.129	0.548
Chains density	2019	0.941	0.874, 1.013	0.107
	2020	0.972	0.892, 1.058	0.509
Chains distance	2019	0.970	0.900, 1.044	0.416
	2020	0.995	0.909, 1.088	0.905
All density	2019	0.969	0.940, 0.999	0.046
	2020	0.975	0.940, 1.011	0.179
All distance	2019	0.911	0.813, 1.020	0.107
	2020	0.914	0.796, 1.050	0.203

A = big chain supermarkets; B = small chain supermarkets & convenience symbol groups; C = independent supermarkets; Chains = A & B; all = A, B & C

Effect estimates of density measures refer to a change in incidence rate in response to an increase of 1 m/km^2. Effect estimates of distance measures refer to a change in incidence rate in response to an increase of 500 m. The reference category for the composition of food environments is neighbourhoods with more supermarkets.

All models are adjusted for age, sex NRS social grade, number of children and adults in the household, region, area deprivation and population density, and interactions between region and NRS social grade, area deprivation, and population density. Note that *p* values have not been adjusted for multiple testing.

3. Including OOH purchases reported from someone other than the main reporter

Table O. Sensitivity analysis of including OOH purchases not reported by the main OOH reporter

		Only from main reporter			All OOH purchases		
Exposure	Year	IR	95% CI	*p* value	IR	95% CI	*p* value
Density of all supermarkets	2019	0.969	0.940, 0.999	0.046	0.968	0.939, 0.998	0.039
	2020	0.975	0.940, 1.011	0.179	0.976	0.941, 1.012	0.186
Distance to any supermarket	2019	0.911	0.813, 1.020	0.107	0.908	0.811, 1.017	0.096
	2020	0.914	0.796, 1.050	0.203	0.926	0.806, 1.064	0.276
Density of restaurants	2019	0.982	0.964, 1.000	0.053	0.981	0.963, 0.999	0.039
	2020	0.992	0.970, 1.014	0.451	0.992	0.971, 1.014	0.484
Distance to restaurants	2019	0.966	0.898, 1.038	0.347	0.971	0.903, 1.044	0.420
	2020	0.990	0.907, 1.080	0.815	0.995	0.911, 1.087	0.911
Density of takeaway outlets	2019	0.987	0.957, 1.018	0.406	0.981	0.951, 1.012	0.221
	2020	0.992	0.956, 1.029	0.653	0.988	0.952, 1.025	0.508
Distance to takeaway outlets	2019	0.957	0.897, 1.021	0.180	0.960	0.900, 1.024	0.215
	2020	0.997	0.921, 1.079	0.938	1.001	0.925, 1.084	0.971
Composition of food environments							
More OOH	2019	0.856	0.620, 1.182	0.344	0.837	0.605, 1.157	0.281
	2020	1.331	0.882, 2.010	0.173	1.186	0.785, 1.793	0.418
No outlets	2019	0.552	0.335, 0.911	0.020	0.611	0.370, 1.009	0.054
	2020	0.810	0.436, 1.505	0.505	0.723	0.387, 1.348	0.307

Effect estimates of density measures refer to a change in incidence rate in response to an increase of 1 m/km^2. Effect estimates of distance measures refer to a change in incidence rate in response to an increase of 500 m. The reference category for the composition of food environments is neighbourhoods with more supermarkets.
All models are adjusted for age, sex NRS social grade, number of children and adults in the household, region, area deprivation and population density, and interactions between region and NRS social grade, area deprivation, and population density. Note that *p* values have not been adjusted for multiple testing.

4. Excluding online food and drink purchases

Table P. Parameter estimates and 95% CI of take-home purchase outcomes associated with food environment exposures, excluding online purchases. n 2019 = 1,201; n 2020 = 1,196

		Frequency			Total Calories			Calories from fruit & vegetables			Calories from HFSS products			Calories from UPF			Alcohol volume		
Exposure	Year	IR	95% CI	*p* value	IR	95% CI	*p* value	IR	95% CI	*p* value	IR	95% CI	*p* value	IR	95% CI	*p* value	IR	95% CI	*p* value
Density of chain supermarkets	2019	1.004	0.987, 1.022	0.850	1.004	0.986, 1.022	0.940	0.997	0.973, 1.022	0.851	1.004	.995, 1.013	0.664	1.003	0.994, 1.011	0.524	0.964	0.857, 1.084	0.827
	2020	1.001	0.984, 1.018	0.967	0.996	0.979, 1.013	0.964	0.980	0.959, 1.002	0.237	1.004	0.996, 1.011	0.766	1.001	0.991, 1.011	0.830	0.962	0.868, 1.066	0.953
Distance to chain supermarkets	2019	0.987	0.970, 1.004	0.338	1.006	0.989, 1.024	0.940	1.013	0.990, 1.037	0.724	0.992	0.983, 1.001	0.598	0.991	0.983, 0.999	0.134	1.032	0.926, 1.151	0.827
	2020	0.976	0.959, 0.994	0.058	0.998	0.981, 1.015	0.964	1.018	0.996, 1.041	0.237	0.998	0.991, 1.005	0.766	0.993	0.983, 1.002	0.306	0.986	0.890, 1.094	0.953
Density of independent supermarkets	2019	1.001	0.992, 1.010	0.850	1.001	0.992, 1.010	0.940	0.998	0.985, 1.011	0.851	0.999	0.994, 1.004	0.806	0.998	0.994, 1.003	0.524	0.968	0.914, 1.026	0.827
	2020	0.996	0.987, 1.005	0.552	0.997	0.988, 1.006	0.964	0.994	0.983, 1.006	0.400	0.999	0.995, 1.003	0.766	0.997	0.992, 1.002	0.406	0.990	0.943, 1.040	0.953
Distance to independent supermarkets	2019	0.993	0.977, 1.010	0.649	1.012	0.995, 1.029	0.940	1.007	0.985, 1.030	0.851	0.997	0.989, 1.006	0.679	0.993	0.985, 1.001	0.213	1.020	0.906, 1.149	0.827
	2020	0.995	0.977, 1.012	0.728	1.009	0.992, 1.026	0.964	1.014	0.991, 1.037	0.315	0.998	0.990, 1.005	0.766	0.994	0.984, 1.004	0.352	1.021	0.919, 1.134	0.953
Density of OOH outlets	2019	1.002	0.999, 1.005	0.397	1.000	0.997, 1.003	0.940	1.000	0.996, 1.004	0.851	1.000	0.998, 1.001	0.920	1.000	0.998, 1.001	0.524	0.997	0.979, 1.015	0.827
	2020	1.002	0.999, 1.005	0.324	0.999	0.996, 1.002	0.964	0.998	0.994, 1.001	0.297	1.000	0.999, 1.001	0.766	0.998	0.997, 0.999	0.306	1.000	0.983, 1.016	0.953
Distance to OOH outlets	2019	0.981	0.961, 1.002	0.296	1.006	0.986, 1.027	0.940	1.023	0.995, 1.051	0.724	0.994	0.984, 1.005	0.598	0.986	0.977, 0.996	0.049	1.023	0.905, 1.156	0.827
	2020	0.983	0.963, 1.002	0.324	0.999	0.979, 1.019	0.964	1.021	0.995, 1.048	0.237	0.995	0.986, 1.003	0.766	0.991	0.979, 1.002	0.306	0.992	0.877, 1.122	0.953
Food environment composition																			
More OOH outlets	2019	0.988	0.913, 1.069	0.850	0.993	0.917, 1.077	0.940	1.029	0.923, 1.147	0.851	0.975	0.935, 1.016	0.598	0.971	0.934, 1.009	0.259	1.056	0.647, 1.723	0.827

	2020	1.002	0.925, 1.085	0.967	0.998	0.921, 1.081	0.964	1.011	0.911, 1.123	0.838	1.006	0.972, 1.042	0.766	0.967	0.924, 1.012	0.306	1.236	0.779, 1.961	0.953
No outlets	2019	0.894	0.791, 1.011	0.296	1.013	0.895, 1.145	0.940	1.118	0.947, 1.319	0.724	0.967	0.907, 1.030	0.598	0.963	0.908, 1.021	0.330	1.275	0.602, 2.700	0.827
	2020	0.913	0.808, 1.032	0.324	0.990	0.877, 1.117	0.964	1.136	0.970, 1.330	0.237	1.018	0.966, 1.072	0.766	0.985	0.920, 1.055	0.758	1.201	0.592, 2.437	0.953

95% CI = 95% confidence interval; HFSS = high in fat, salt and sugar; IR = Incidence Rate; OOH = out of home; UPF = ultra-processed foods. Effect estimates of density measures refer to a change in incidence rate in response to an increase of 1 m/km^2. Effect estimates of distance measures refer to a change in incidence rate in response to an increase of 500 m. The reference category for the composition of food environments is neighbourhoods with more supermarkets.

All models are adjusted for age, sex and social grade of the main shopper, number of children and adults in the household, region, area deprivation and population density, and interactions between region and social grade, area deprivation, and population density. *p* values were adjusted for multiple testing using the Benjamini-Hochberg method.
